# Lifestyle, environment and other major determinants of frailty in older adults: a population-based study from the UK Biobank

**DOI:** 10.1007/s10522-025-10242-x

**Published:** 2025-05-03

**Authors:** Ali Hemadeh, Carlota Lema-Arranz, Stefano Bonassi, Leonardo Buscarini, Francesco Infarinato, Paola Romano, Alessia Finti, Franco Marinozzi, Fabiano Bini, Natalia Fernández-Bertólez, João Paulo Teixeira, Laura Lorenzo-López, Vanessa Valdiglesias, Blanca Laffon

**Affiliations:** 1 Grupo DICOMOSA, Departamento de Psicología, CICA—Centro Interdisciplinar de Química e Bioloxía, Universidade da Coruña, A Coruña, Spain; 2 Instituto de Investigación Biomédica de A Coruña (INIBIC), Complexo Hospitalario Universitario de A Coruña (CHUAC), Sergas, A Coruña, Spain; 3 Unit of Clinical and Molecular Epidemiology, IRCCS San Raffaele Roma, Rome, Italy; 4 Department of Human Sciences and Quality of Life Promotion, San Raffaele University, Rome, Italy; 5 Rehabilitation Bioengineering Laboratory, IRCCS San Raffaele Roma, Rome, Italy; 6 Department of Mechanical and Aerospace Engineering (DIMA), Sapienza University of Rome, Rome, Italy; 7 Grupo NanoToxGen, Departamento de Biología, CICA—Centro Interdisciplinar de Química e Bioloxía, Universidade da Coruña, A Coruña, Spain; 8 Environmental Health Department, National Institute of Health Doutor Ricardo Jorge, Porto, Portugal; 9 EPIUnit—Instituto de Saúde Pública, Universidade do Porto, Porto, Portugal; 10 Laboratory for Integrative and Translational Research in Population Health (ITR), Porto, Portugal; 11 Gerontology and Geriatrics Research Group, Instituto de Investigación Biomédica de A Coruña (INIBIC), Complexo Hospitalario Universitario de A Coruña (CHUAC), Sergas, Universidade da Coruña, A Coruña, Spain

**Keywords:** Frailty, Lifestyle, Environment, Principal component analysis, Machine learning

## Abstract

Frailty is a geriatric multidimensional syndrome characterized by a loss of physiologic reserves and disproportionate vulnerability to external stressors and associated with increased risk of multiple negative health outcomes. Since frailty can be prevented, controlled, and even reverted in its early stages, identifying the main factors involved in its development is crucial to implement preventive and/or restorative interventions. The aim of this study was to assess the impact of a broad range of parameters, including host factors, lifestyle, diet, and environmental and occupational conditions, on the development of frailty in later life. A cross-sectional study was conducted on 221,896 individuals aged 60 and over classified as non-frail (119,332, 53.8%), pre-frail (93,180, 42.0%), and frail (9384, 4.2%) according to the frailty phenotype. Using principal component analysis and machine learning to streamline the data, significant associations were found between frailty risk and air quality, diet, smoking, working conditions, and heavy alcohol consumption. Early-life factors, including breastfed as a baby and maternal smoking around birth, also emerged as predictors of frailty, which was further characterized by clinical indicators like polypharmacy, levels of C-reactive protein and other biomarkers of inflammageing. This study provided robust and original evidence on the association between a large battery of potential risk factors, from early to later stages of life, and the occurrence of frailty in older age. These results will contribute to the development of effective prevention strategies and facilitate the early detection of individuals at high risk of developing frailty.

**Supplementary Information:**

The online version contains supplementary material available at 10.1007/s10522-025-10242-x.

## Introduction

The global population is experiencing a significant increase in the number and proportion of older adults. It is expected that the number of people over 60 years old increases from 1 billion in 2019 to more than 2.1 billion by 2050 (WHO 2024). These demographic changes will shape future healthcare use and healthcare expenditures (European Commission 2015).

Ageing can be defined as the time-related deterioration of the physiological functions necessary for survival and fertility (Gilbert 2000). Hence, ageing is a natural, gradual, and continuous process. In frailty, this deterioration is accelerated and accompanied by failure of homeostatic mechanisms (Clegg et al. 2013); it is therefore considered as “unsuccessful ageing”. Frailty is considered a geriatric multidimensional syndrome characterized by a loss of physiologic reserves and disproportionate vulnerability to external stressors (Dent et al. 2019). Frailty has been linked to an increased risk of negative health outcomes in older adults, including falls, injuries, prolonged hospital stays, institutionalization, loss of independence, and higher mortality rate (Vermeiren et al. 2016; Hoogendijk et al. 2019), leading to a high demand of care and reduced quality of life. Indeed, several recent meta-analyses reported the association of frailty with an increased risk of all-cause mortality, and cause-specific mortality from cardiovascular disease, cancer, and respiratory illness (Kojima et al. 2018b; Yang et al. 2018; Peng et al. 2022). Thus, frailty is not an inevitable consequence of the ageing process; rather, frailty can be viewed as an accelerated or pathological state of ageing (Ye et al. 2023).

A wide variety of tools is available to identify frailty. One of the simplest and most frequently used in clinics is the Fried’s phenotype criteria, which defines frailty on the basis of the presence of at least three of the following indicators: unintentional weight loss, fatigue, slow walking pace, muscle weakness, and low physical activity (Fried et al. 2001), thus facilitating applicability and reproducibility (Livshits et al. 2018). A different approach, also broadly used, is the cumulative model proposed by Rockwood and Mitniski (Mitnitski et al. 2001), that calculates the Frailty Index by quantifying the total burden of health deficits across multiple clinical domains (e.g., diagnoses, disability, cognitive function, and physical function) as a proportion.

The prevalence of frailty greatly varies across studies and depends on the frailty identification tool used. A recent meta-analysis including participants from 62 countries reported that the prevalence of frailty was 12% using physical frailty measures and 24% using the frailty index (O’Caoimh et al. 2021). Another contemporary meta-analysis including 57 studies from Western countries revealed that around 26.8% of the older population have a diagnosis of frailty, evaluated using the multidimensional prognostic index (MPI, a tool for clinical decision making able to capture different aspects of frailty condition according to multidimensional model) (Veronese et al. 2021). Anyway, due to the rapid population ageing, frailty is receiving growing attention worldwide as it imposes a significant burden on healthcare systems and the economy (Chi et al. 2021). Extensive research is underway to identify mechanistic pathways and preventable risk factors associated with frailty.

Frailty per se is emerging as a prime candidate for disability prevention in the modern era of function-centered medicine. Identifying the main factors involved in frailty development is crucial to implement preventive and/or restorative interventions to improve quality of life and independency of older adults, even more considering that frailty is a dynamic process that worsens or improves over time, and its negative effects can be prevented, controlled, and even reverted in its early stages (Rodriguez-Mañas and Fried 2015). Indeed, older populations were consistently found to have dynamic and bidirectional changes in their frailty state (robust, pre-frail, and frail) in a meta-analysis by Kojima et al. (2019b).

**Fig. 1:**
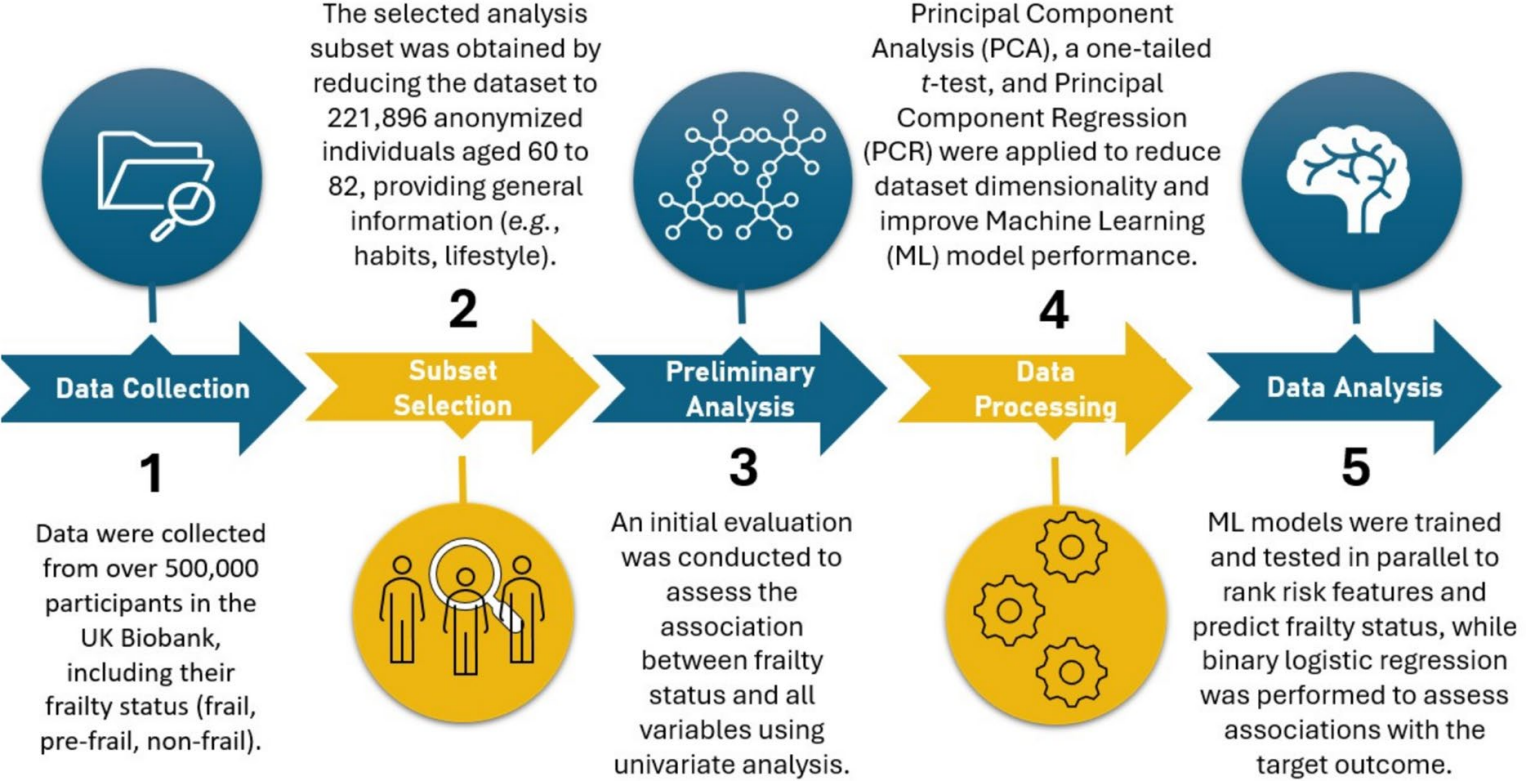
Flow of statistical/bioinformatic analyses

The primary aim of this study was to assess the impact of a broad range of parameters, including host factors, lifestyle, diet, and environmental and occupational conditions, on the development of frailty in later life. Additionally, the analysis incorporated data from a set of biomarkers linked to early or intermediate stages of major chronic diseases, as well as selected parameters describing pre- and perinatal conditions. A cross-sectional study was therefore conducted in a large cohort of UK biobank participants aged 60 and over, who were classified according to their frailty status by means of the phenotype criteria (Fried et al. 2001). To manage the complexity of the statistical analysis, which involved over 50 variables across 221,896 subjects, a series of advanced data mining techniques were employed to reduce the dimensionality of the dataset. These included principal component analysis (PCA) and machine learning (ML), as outlined in Fig. 1 and thoroughly detailed in the methods section.

## Methods

### Study cohort

For this study, we used data from the UK Biobank (https://www.ukbiobank.ac.uk/), a large-scale biomedical database and research resource containing health, lifestyle and genetic information from approximately 500,000 adult participants, aged 37 to 73 years old in the initial evaluation, recruited in 2006–2010 from across the United Kingdom. All participants gave written informed consent for data collection, analysis, and linkage. Ethics approval for the UK Biobank study was obtained from the North West Multi-centre Research Ethics Committee (11/NW/0382). The analyses reported in this manuscript are part of UK Biobank project 54032. For the purpose of this study, only participants aged 60 years or older were included. During the initial assessment, participants completed a touchscreen questionnaire, a nurse-led interview, underwent physical and functional measurements, and provided blood, urine, and saliva samples. Participants also consented to have their future health events monitored. While all participants attended an initial assessment center (instance 0, between 2006 and 2010), a subset was invited to return several years later to repeat certain assessments during subsequent instances: instance 1 (2012–2013), instance 2 (from 2014), and instance 3 (from 2019). Further details on UK Biobank have been previously described (Sudlow et al. 2015).

### Frailty status in the UK Biobank

Frailty status was assessed using the five frailty phenotype criteria originally defined by Fried et al. (2001). However, as the specific questions and measurements collected in the UK Biobank differed from those in the original Cardiovascular Health Study (CHS), we applied the adaptations outlined by Hanlon et al. (2018), with the exception of the grip strength cut-offs, which were adjusted to account for the considerable differences in grip strength ranges between the CHS and UK Biobank cohorts. To ensure accuracy, we used sex-specific cut-offs (calculated for individuals aged 60 and over), defining the lowest quintile as indicative of low grip strength, as recommended by Fried et al. (2001). A detailed description of how the other four Fried criteria were adapted to data available in the UK biobank is reported in the paper mentioned above (Hanlon et al. 2018).

Participants were categorized as non-frail (negative for all frailty criteria), pre-frail (positive for one or two criteria), or frail (positive for three or more criteria). Individuals with missing data for one or more frailty indicators were excluded from the analysis.

Since the evaluation process in the UK Biobank occurred across four consecutive instances (0 to 3), we used data from the most recent instance available for each individual in which all parameters necessary to assess frailty status were measured. This approach ensured that participants were assessed at their oldest possible age, thereby increasing both the total number of participants and the number of frail individuals included, compared to using data from the initial instance. For most variables evaluated in this study, data were available from the four instances, so we used data from the same instance where frailty status was determined. For some variables data were available from just instance 0 (e.g. air pollution estimates, work environment parameters).

### Lifestyle, diet, and other parameters included in the statistical analysis

#### Lifestyle and diet

The following variables were studied for their possible association with frailty:

Alcohol consumption: Alcohol drinker status (categorized as never, previous, current). Alcohol intake frequency (daily or almost daily, 3–4 times/week, 1–2 times/week, 1–3 times/month, special occasions only, never). Alcohol intake compared to 10 years ago (more nowadays, about the same, less nowadays). Frequency of consuming six or more units of alcohol (a “drink” is defined as one unit of alcohol) (never, less than monthly, monthly, weekly, daily or almost daily).

Exposure to tobacco smoke: Smoking status (never, previous, current). Pack-years (number of cigarettes smoked in the lifetime, resulting from the multiplication of the number of packs smoked per day by the number of years a person has smoked). Exposure to tobacco smoke: this variable considers exposure to the own tobacco smoke for current smokers, and secondhand smoke exposure for non-smokers. It was calculated by combining the variables Exposure to tobacco smoke at home, Exposure to tobacco smoke outside home, and Smoking status; the former two variables were collected only from participants who did not indicate they currently smoke tobacco on most or all days, so they reflect secondhand smoke exposure in non-smokers (never or previous smokers).

Diet: Cooked vegetable intake, and Salad/raw vegetable intake (both measured as tablespoons/day). Fresh fruit intake (pieces/day). Oily fish intake, Non-oily fish intake, Processed meat intake, Poultry intake, Beef intake, Lamb/mutton intake, Pork intake, and Cheese intake (all categorized as never, less than once a week, 2–4 times/week, 5–6 times/week, once or more daily). Milk intake: this variable was calculated by recoding the variable Milk type used (categorized as yes = full cream, semi-skimmed, skimmed, or never/rarely have milk = never/rarely, soya, other type of milk). Bread intake (slices/week). Cereal intake (bowls/week). Salt added to food (not including salt used in cooking, categorized as never/rarely, sometimes, usually, always). Tea intake (cups/day). Coffee intake: this variable was calculated by recoding the variable Coffee type (categorized as no = decaffeinated coffee, or yes = instant coffee, ground coffee, other type of coffee). Water intake (glasses/day). Major dietary changes in the last 5 years (no, yes because of illness, yes because of other reasons).

Dietary supplements: Vitamin and mineral supplements (this variable considers vitamins A, B, C, D, E, B9, and multivitamins/minerals; it was recoded in yes/no). Mineral and other dietary supplements (considering fish oil, glucosamine, calcium, zinc, iron, and selenium; it was recoded in yes/no).

#### Quality of the environment

Natural environment %: The percentage of the home location buffer classed as ‘Natural Environment’ in the Land Cover Map (LCM) 2007, and with home location data buffered at 1000 m. Land use data were obtained from the LCM 2007, and the 23 land cover classes were reclassified to a binary classification; classes 1–2 (‘Natural Environment’), and 22–23 (‘Built environment’).

Air pollution in the UK Biobank was defined using a Land Use Regression (LUR) model developed as part of the European Study of Cohorts for Air Pollution Effects (ESCAPE) project (http://www.escapeproject.eu/) (Beelen et al. 2013). The LUR model is based on ESCAPE monitoring done between 26 Jan 2010 and 18 January 2011, and air pollution estimates are representative for the year 2010 (initial instance). Air pollution estimates were modelled for each participant’s geocoded address. Since ESCAPE estimates for particulates are valid up to 400 km from the monitoring area (Greater London), but it is unclear how good the estimates are outside this area, addresses located more than 400 km away from Greater London were not assigned PM10 and PM2.5 concentrations (missing data).

#### Work environment

Forty arrays were available for all these variables, in only one instance. All of them were categorized as rarely/never, sometimes/often. When there was an answer “sometimes/often” in any of the 40 arrays, the final answer was coded as “sometimes/often”. When there was an answer “rarely/never” in any of the 40 arrays, and no answers “sometimes/often”, the final answer was coded as “rarely/never”. The variables analysed were: Workplace very noisy, Workplace very cold, Workplace very hot, Workplace very dusty, Workplace full of chemical or other fumes, Worked with materials containing asbestos, Worked with paints, thinners or glues, Worked with pesticides, and Workplace had a lot of diesel exhaust.

#### Other potential predictors of frailty

Use of sun/UV protection (categorized as never/rarely, sometimes, most of the time, always, do not go out in sunshine). Frequency of solarium/sunlamp use: this variable was recoded in never, up to 10 times/year, more than 10 times/year.

Polypharmacy: was calculated recoding the variable Number of treatments/medications taken (categorized as no polypharmacy ≤ 5 medications, or polypharmacy = 5 or more medications).

Early life factors: Breastfed as a baby (yes/no). Maternal smoking around birth (yes/no). Data for these two variables were taken from instance 0.

#### Biomarkers

C-reactive protein (CRP) (mg/L), insulin-like growth factor 1 (IGF-1) (nmol/L), oestradiol (pmol/L), rheumatoid factor (IU/mL), sex hormone binding globulin (SHBG) (nmol/L), testosterone (nmol/L) and vitamin D (nmol/L).

A list of notations and acronyms used for the parameters included in the analyses is reported in Supplementary Table 1.

### Statistical analysis

Given the complexity of the dataset, a step-by-step approach was implemented to reduce the dimensionality of the analysis. First, a basic univariate analysis was conducted to evaluate the association between potential predictors and frailty status (three levels), using ANOVA for continuous variables and chi-square tests for categorical variables. However, to manage the large number of variables collected by the UK Biobank, we applied Principal Component Analysis (PCA) to reduce the dimensionality and identify the variables most strongly associated with frailty. The resulting principal components were then analyzed using Principal Component Regression and further refined by assessing feature importance through Machine Learning (ML) techniques. The final step of the analysis involved a classic logistic regression to estimate Odds Ratios (ORs) for the variables selected in the previous multidimensional analyses. For simplicity, the final results focus solely on the risk of frailty, as the analyses for pre-frailty systematically yielded intermediate ORs between those of the frailty group. The analysis workflow is provided in Fig. 1.

#### Principal component analysis (PCA)

PCA was applied to a dataset of non-frail, pre-frail, and frail patients over 60 to identify a set of questions capable of predicting frailty status. Prior to analysis, Z-score standardization was used to transform each variable by subtracting its mean and dividing by its standard deviation, ensuring comparability across continuous and categorical variables without further treatment. The goal of the analysis was to uncover hidden relationships among the original questionnaire variables and reduce the number of parameters needed for frailty assessment. By focusing on the most influential variables in terms of explained variance, the analysis aimed to streamline the evaluation process while retaining the most predictive relationships. The interpretation of PCA outputs (Supplementary Table 2) focused on identifying variables most strongly correlated with frailty status across different principal components (PC). The frailty phenotype, which categorizes patients into non-frail, pre-frail, and frail groups, was included in the analysis alongside other variables. An Independent Samples *t*-test was performed to determine whether the PC showed statistically significant differences between frailty classes (Tsuchida et al. 2022). Components where the frailty status had a statistically higher weight compared to its contribution in other PC were considered more representative of frailty. To identify a reduced set of questions that were both necessary and sufficient to describe frailty status, the correlation between the frailty variable and other variables was analyzed. In PCA biplots, the cosine of the angle between vectors representing variables corresponds to their correlation coefficient (Jolliffe and Cadima 2016). Therefore, the correlation analysis was conducted in angular terms (see Supplementary Material 1). An angle close to zero indicates a strong positive correlation (coefficient of 1), an angle near 90° indicates no correlation (coefficient of 0), and an angle close to 180° reflects a strong inverse correlation (coefficient of − 1) (Kohler and Luniak 2005). To further reduce dimensionality and address multicollinearity, a subset of PC explaining the most variance was selected for use as independent variables in a linear regression model to predict the outcome. All analyses were performed using the MATLAB environment.

#### Machine learning analysis and features importance

The dataset for the machine learning (ML) analysis includes 18 variables: one categorical target variable and 17 predictors derived from PCA results. Using label encoding, individuals were assigned to three classes: 0 for non-frail, 1 for pre-frail, and 2 for frail. Missing values were imputed using the mean for quantitative variables and the most frequent value for categorical ones, following the approach of Loladze et al. (2024), and implemented with Python’s scikit-learn library (Pedregosa et al. 2011). Continuous variables were scaled using the MinMaxScaler to standardize their distribution and address outliers, ensuring equal weight across features, as demonstrated by Ozkara et al. (2023).

To handle target class imbalance, we applied balancing techniques including Random Over/Under Sampling (ROS/RUS), SMOTE, ADASYN, SMOTE-Tomek, and Cluster Centroids using the imbalanced-learn library. Classifiers such as LightGBM, Gradient Boosting, Random Forest, and XGBoost were tested, with models like LightGBM being identified as optimal for frailty prediction in prior research by Mizuguchi et al. (2024). Feature importance was assessed using scikit-learn tools to rank variables and reduce dimensionality, enhancing prediction accuracy.

After PCA, the dataset was split into training (80%) and test sets (20%). The importance ranking of PCA-derived variables informed the weights in the ML models. Finally, we evaluated LightGBM’s performance on the test set, predicting frailty classes based on diverse variables, including diet, lifestyle, and environmental indicators.

## Results

In this study, data from 221,896 individuals aged from 60 to 82 from the UK Biobank were analyzed. Among them, 119,332 (53.8%) were classified as non-frail, 93,180 (42.0%) as pre-frail, and 9384 (4.2%) as frail. The typical frail patient is more often female (55.3%) with a mean age of 64.9 ± 3.6 years, although the censoring of the UK biobank to 82 years of age, makes these figures applicable only to this age range. A comprehensive univariate association between all the variables considered in the analysis and the three categories of frailty is reported in the Supplementary Table 3. The large numbers involved make the statistical comparisons poorly informative since almost all variables were associated with frailty status with a highly significant P value. Informative clues come from the distribution of mean values and proportions. To reduce the dimensionality of the dataset, the PCA was applied to transform this large set of correlated variables into uncorrelated PC. These PC are linear combinations of the original variables, ordered by the amount of variance they explain.

### Principal component analysis and principal component regression

**Fig. 2:**
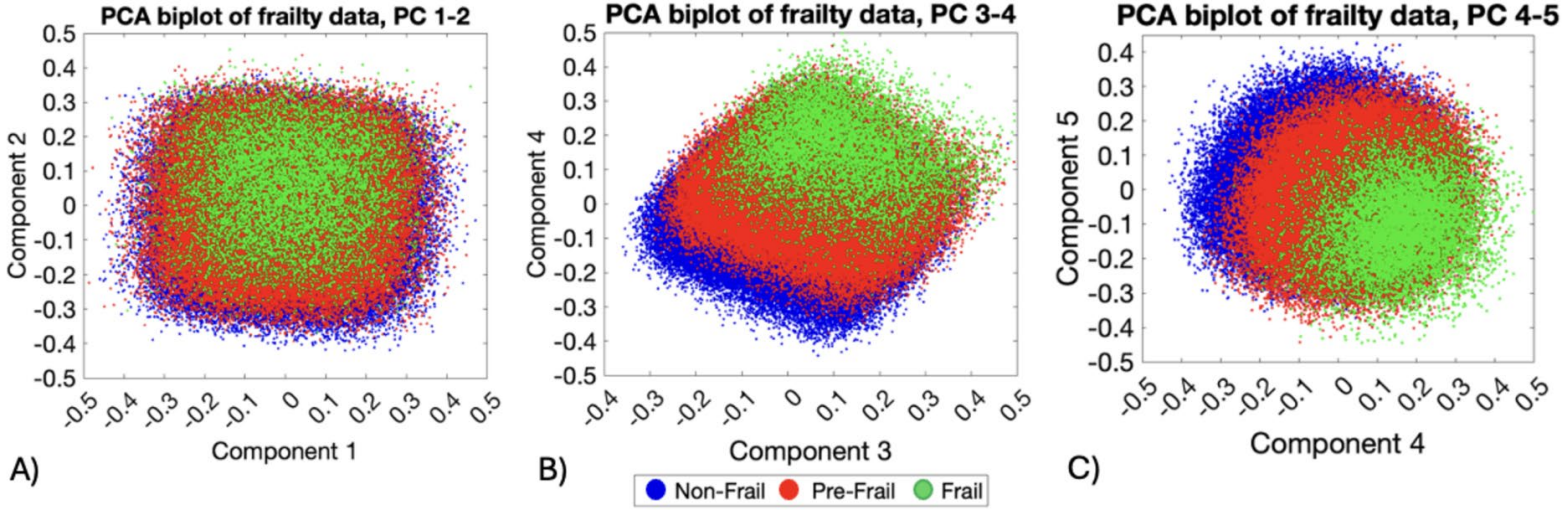
PCA biplots illustrating the separation of frailty classes along the first five principal components (PC): **A** PC1 vs. PC2, **B** PC3 vs. PC4, and **C** PC4 vs. PC5

**Fig. 3:**
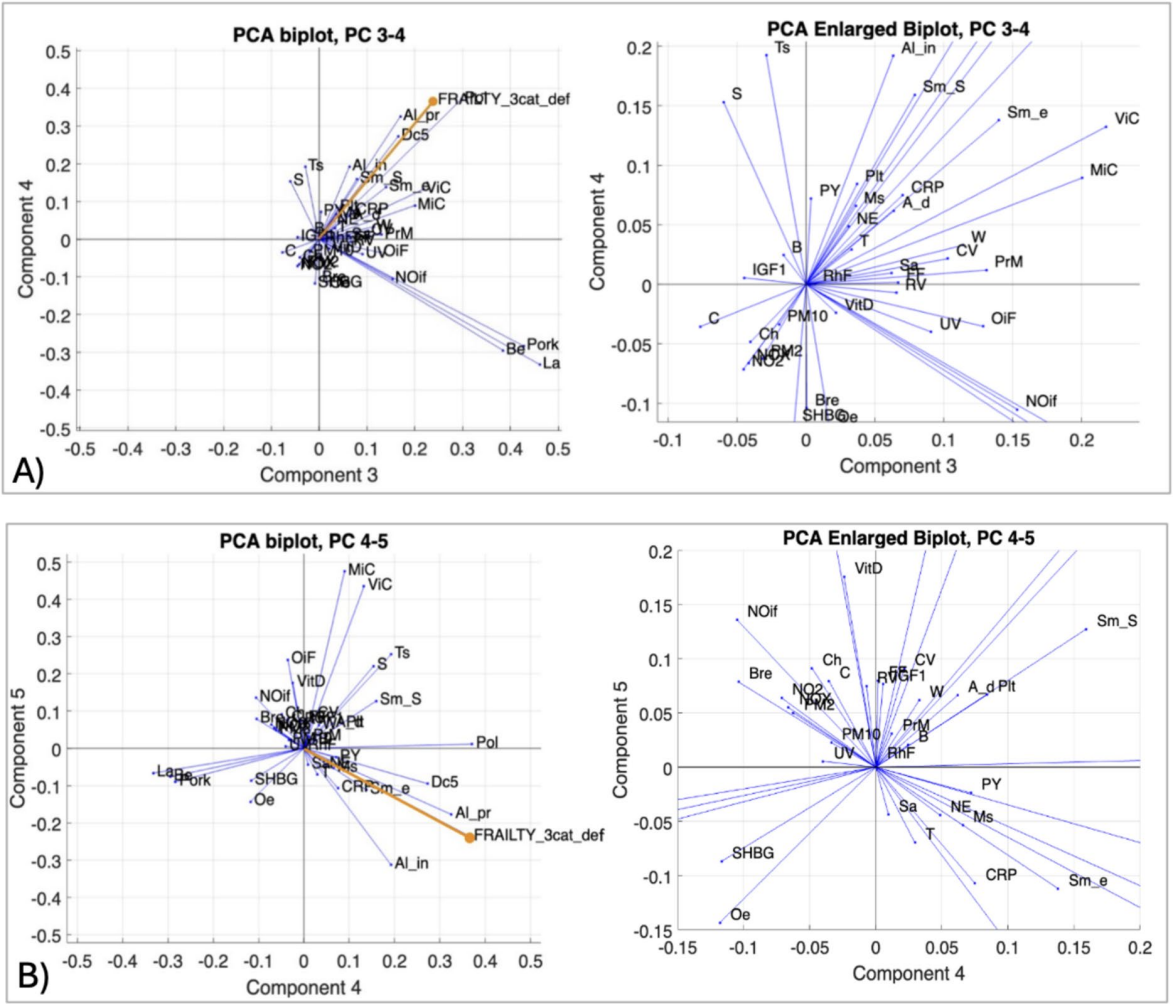
PCA biplots describing the distribution of variables across Principal Components (PC) 3 and 4 (**A**) and PC 4 and 5 (**B**). The full PCA biplots (left panels) show the arrangement of variables relative to frailty status (FRAILTY_3cat_def), indicated by an orange vector. The right panels provide enlarged views of the PCA biplots. See Supplementary Table 1 for notation and acronym explanations

**Fig. 4:**
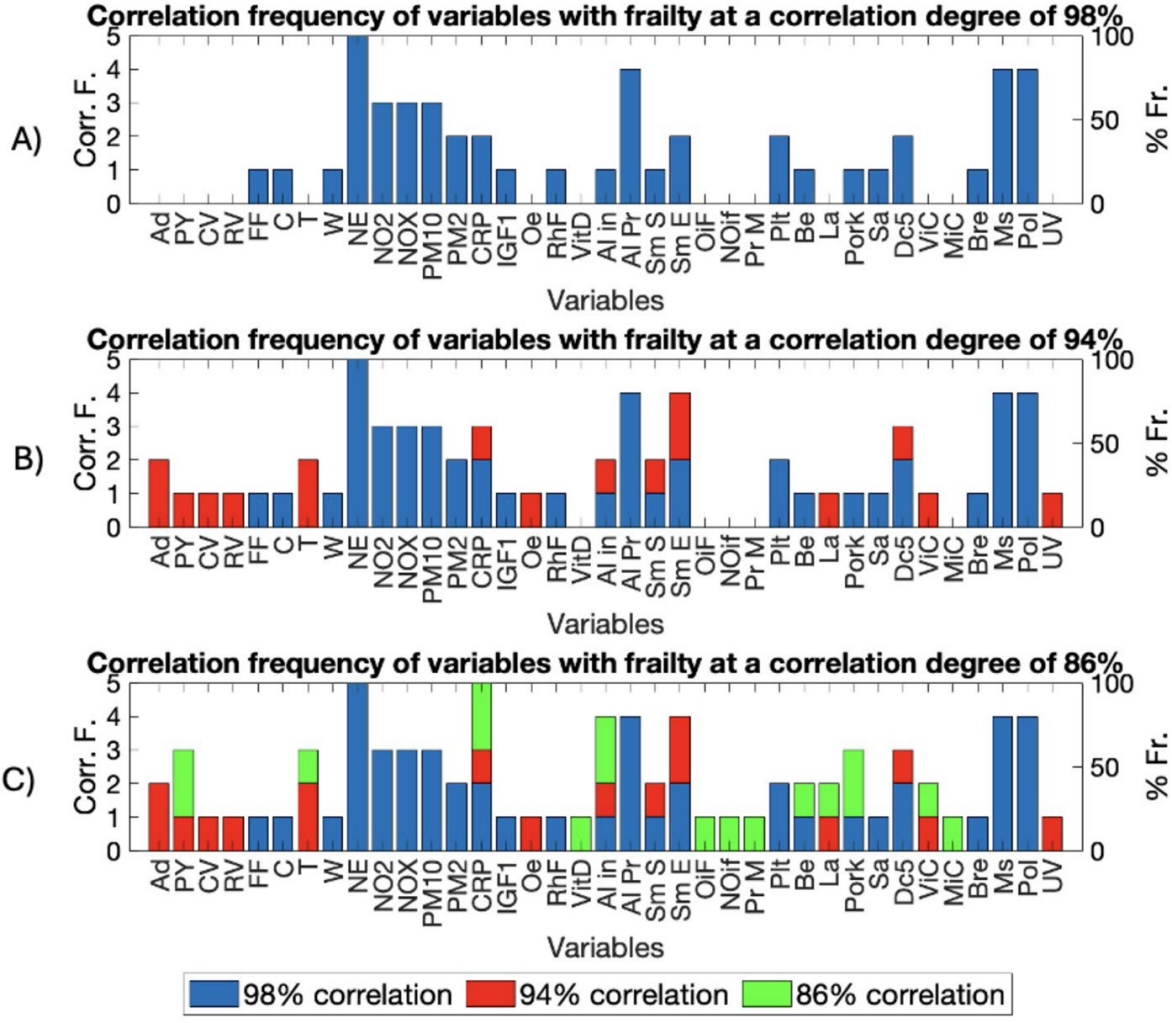
Bar charts indicating the frequency with which each variable relates to frailty status across the first seven principal components, evaluated at different correlation thresholds. **A** Correlations ≥ 98%; **B** correlations ≥ 94%; **C** correlations ≥ 86%. Corr. F.: correlation frequency; % Fr.: % frequency. See Supplementary Table 1 for notation and acronym explanations

Using the “*elbow*” criterion applied to the scree plot, seven initial PC were selected, ordered by the amount of variance they captured from the data (Supplementary Table 4). After verifying the significant separation between the first seven PC, a right-tailed *t*-test on the loadings of the variable ‘Frailty status’ identified PC3, PC4, and PC5 as the most representative components for frailty (h = 1, P value = 0.0012). The separation of classes along the first five PC is reported in Fig. 2. PCA biplots of the frailty data considering PC1 and PC2, although explain most of the variance, do not show a clear separation between the classes of non-frail, pre-frail, and frail subjects. In contrast, the PCA biplots PC3/PC4 and PC4/PC5 show a clear separation between the subject classes, indicating that these components are more representative of frailty. Variables with progressive strength of correlation, i.e., 86%, 94%, and 98%, for biplots PC3/PC4 and PC4/PC5 were considered as relevant (indirectly correlated variables were included). Figure 3 presents the biplots for variables included in the PC3/PC4 and PC4/PC5, while the Supplementary Table 5 shows the list of variables directly and indirectly correlated to the frailty status according to correlation evaluated by parametric angular ranges. Finally, the frequency with which each variable correlated with frailty status across the first seven PC was calculated. Bar charts reported in Fig. 4 show those variables that appeared to be more frequently related to the frailty status across the first seven PC. The results include variables correlated at least once with an 86%, 94%, or 98% correlation degree. The most frequently correlated variables with frailty status were Natural environment % and CRP, followed by Alcohol intake frequency, Alcohol intake compared to 10 years ago, Exposure to tobacco smoke, Maternal smoking around birth, and Polypharmacy. These variables accounted for the 37% of the total ≥ 86% correlations (n = 81). Variables with at least three correlations ≥ 86% included Pack-years, Tea consumption, Pork consumption, and Major dietary changes in the last 5 years. Overall, 33 variables were correlated with frailty status at least once in one of the seven PC investigated. The list included: Age at assessment of frailty, Smoking status, Poultry, Beef, and Lamb intake, and Vitamin and mineral supplements, and at a lower degree of correlation Cooked vegetable, Raw vegetable, Fresh fruit, Cereal, Water, Oily fish, Non-oil fish, and Processed meat intake, Salt added to food, the levels of IGF-1, Oestradiol, Rheumatoid Factor, Vitamin D, and eventually, Breastfed as a baby, and Use of sun/UV protection.

To further reduce the dimensionality of the models, and to improve model stability, we applied the PC regression to identify uncorrelated PC and select the most informative. The first 7 PC explaining the majority of the variance were selected, while the remaining components were discarded to avoid overfitting. Importantly, the PCA was performed excluding the variable representing frailty status to allow its use as the independent variable in the regression analysis (Supplementary Table 6). After fitting the model, the coefficient β corresponding the PC5 was by large the most informative (Supplementary Fig. 1). The loadings of the original variables contributing to this PC were estimated (Supplementary Table 7).

### Machine learning and features ranking

**Fig. 5:**
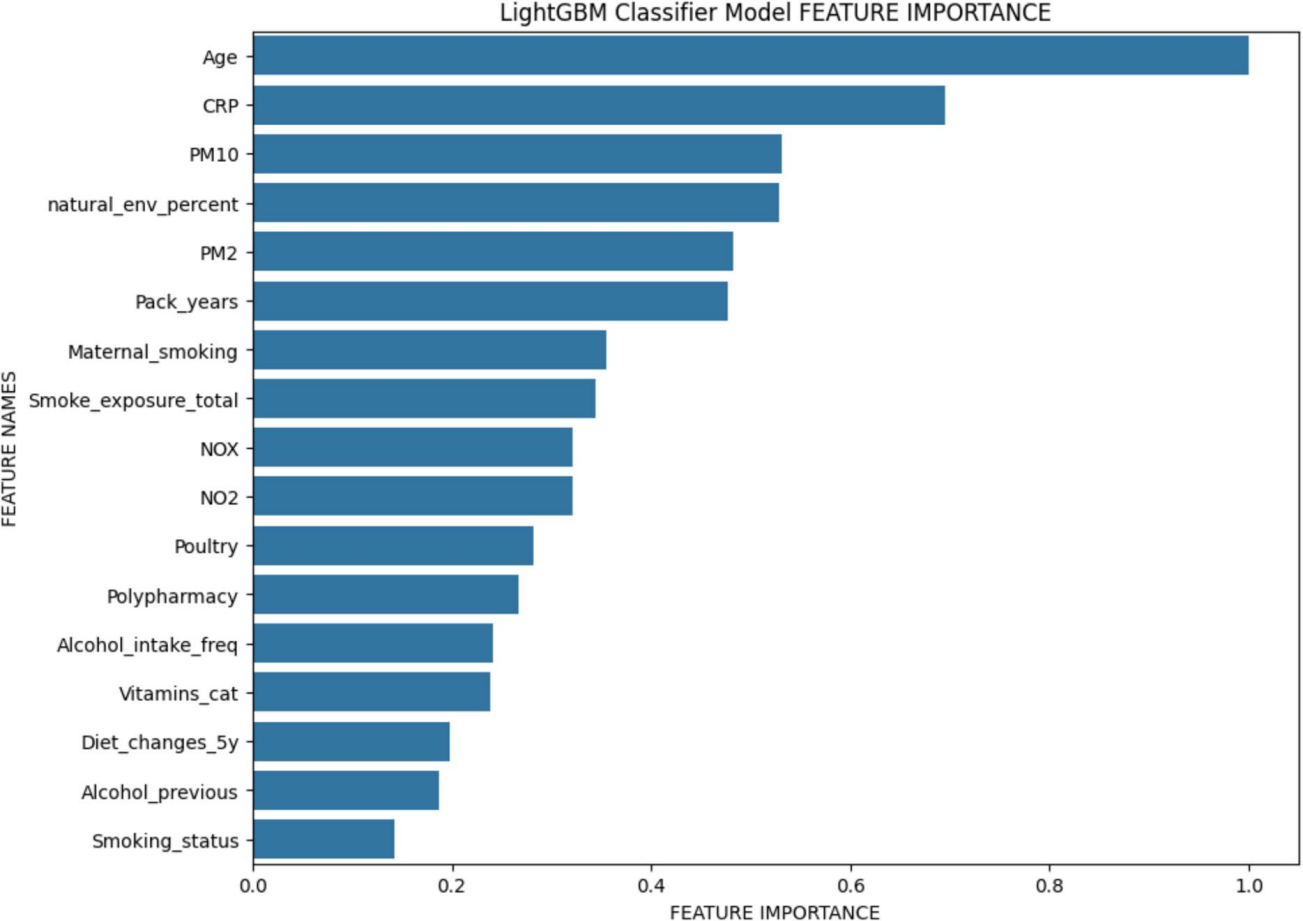
Ranking of features impact in predicting frailty status identified through Light Gradient Boosting Machine (LGBM). See Supplementary Table 1 for notation and acronym explanations

The dataset used for machine learning (ML) analysis comprises 221,896 anonymized observations. It includes 18 variables (features), consisting of one categorical target variable and 17 predictor variables derived from PCA results encompassing both categorical and quantitative data. The accuracies reported by each balancing technique and classifier were compared for the training and test sets. As shown in the Supplementary Table 8, Light GBM (LGBM) emerges as the top-performing model when Cluster Centroids (CC) is employed as the balancing technique. It achieved 98% accuracy on the Training Set (80% of the original dataset) and 94% on the Test Set (20% of the original dataset) in terms of model prediction accuracy. The performance of the LGBM in predicting the target variable (frailty/pre-frailty/non-frailty), emphasizing the ranking of PCA-derived features to which the model assigns higher importance, is reported in Fig. 5. Age was by large the top predictor of frailty status, despite the short age range which did not include subjects ≥ 82 years old. CRP ranked 2nd out of 17 variables included in the ML analysis. Within the first 10 variables for feature importance in predicting frailty or pre-frailty, 5 of them were related to the poor quality of the living environment, in terms of air pollution markers, such as PM10 (particulate matter < 10 µm in diameter), PM2.5 (particulate matter < 2.5 µm in diameter), NO_X_ (nitrogen oxides), NO_2_ (nitrogen dioxide), or considering the residence in a built environment, in contrast with a natural environment. Exposure to tobacco smoke was also among the variables most strictly associated with the frailty status, expressed as pack-years, or as overall exposure to tobacco smoke. Early life factors played also a role placing Maternal smoking around birth among the most important features. Other parameters to be considered, though to a lower extent, were the presence of polypharmacy and dietary features, including alcohol.

### Binary logistic regression of individual variables

To provide a quantitative estimate of the association between the variables selected by PCA and ML with frailty, a binary logistic regression analysis was conducted within homogeneous exposure groups. In all analyses, the group of pre-frail showed intermediate results with respect to frail subjects, so for a more thorough presentation only results from the comparison of frail vs. non-frail are presented. All logistic regression analyses included the variables identified through dimensionality reduction techniques. In addition to this core set, specific variables related to the type of exposure under investigation were also included. Some results were intentionally reported without referencing a table or figure to focus only on findings that warranted further consideration. All these results were obtained from similar logistic regression models that included actual confounders selected from the set of variables identified through dimensionality reduction.

#### Alcohol intake

The univariate analysis of the association between frailty status and alcohol consumption shows clearly that frail patients tend to be less likely alcohol drinkers, with a frequency of abstainers nearly double when compared to non-frail subjects (9.8% vs. 4.3%; P < 0.0001). To investigate this inverse relationship in more detail, we estimated the risk of being frail with different drinking patterns, to identify a dose–response curve. Actually, the risk of being frail is significantly reduced in those drinking daily or almost daily versus abstainers (OR = 0.207, 95%CI 0.0192–0.225, P < 0.0001). The lower risk associated with drinking constantly decreases up to those drinking only occasionally. In this latter group, the reduction is still present though smaller (OR = 0.741, 95%CI 0.692–0.793, P < 0.0001). To implement data on drinking frequency with data on consumption, we investigated the effect of heavy drinking (Table 1). Subjects occasionally (monthly or less) drinking 6 or more units of alcohol still have a lower risk of being frail, while this reduction disappears for weekly heavy drinking and reverses for those drinking 6 units of alcohol or more every day (OR = 1.358, 95%CI 1,011–1,823, P < 0.05). As regards the change of drinking habit, frail subjects show a significantly higher tendency to reduce drinking in the last ten years (64.5% vs. 44.4%, P < 0.0001).

**Table 1:** Binary logistic regression analysis of high alcohol intake (> 6 Units) and frailty status

Alcohol intake (> 6 Units)	β	SE	P value	OR	95%CI
Never				1.00	–
Less than monthly	− 0.334	0.089	0.001	0.716	0.601–0.852
Monthly	− 0.343	0.142	0.016	0.710	0.537–0.938
Weekly	− 0.136	0.113	0.230	0.873	0.699–1.090
Daily or almost daily	0.306	0.150	0.042	1.358	1.011–1.823

Estimates adjusted for sex, age at the assessment of frailty, natural environment %, C-reactive protein (mg/L), and smoking status

*β* regression coefficient, *SE* standard error, *OR* odds ratio, *95%CI* 95% confidence interval

#### Smoking habit

**Fig. 6:**
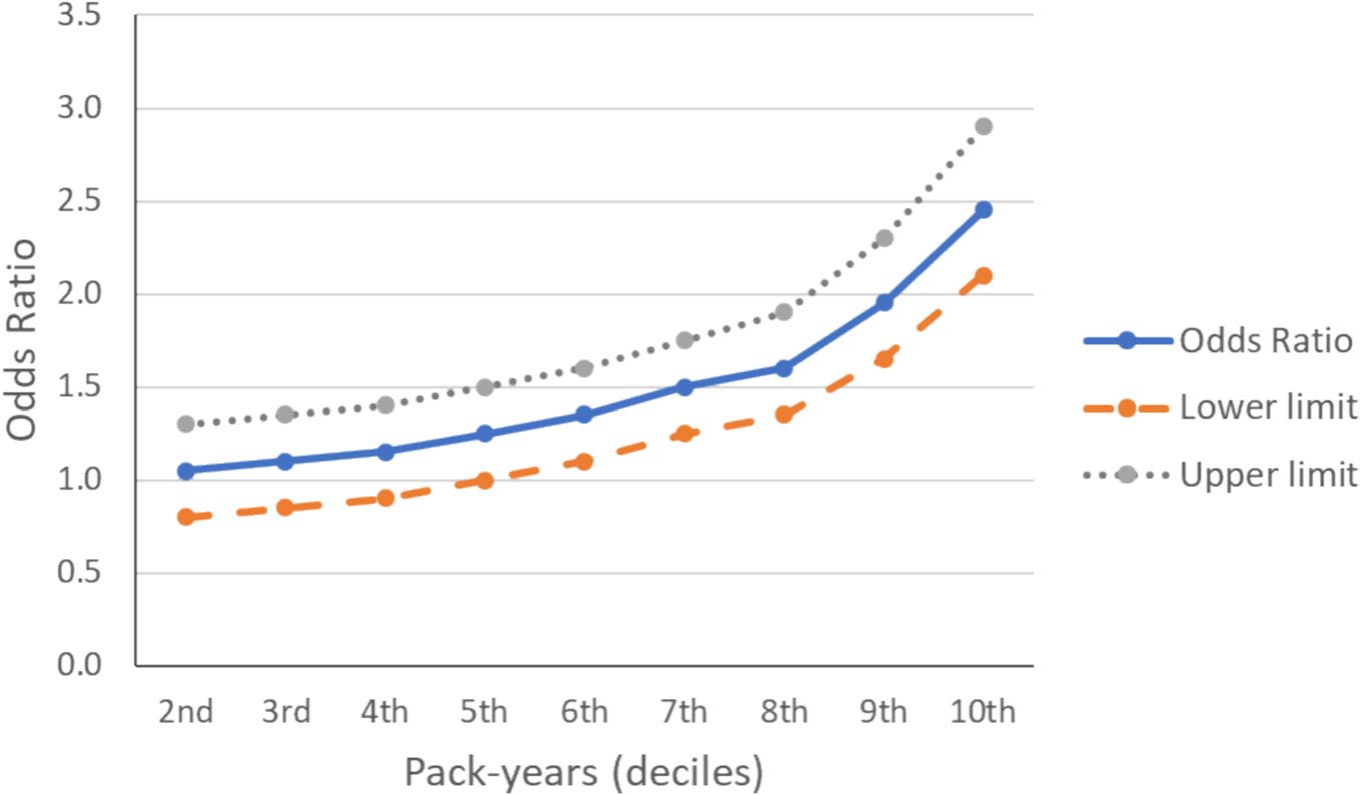
Binary logistic regression of percentile of cigarette consumption (pack-years) and frailty status. Estimates adjusted for sex, age at the assessment of frailty, C-reactive protein (mg/L), alcohol intake frequency, and natural environment %

The positive and consistent association between frailty and smoking habit is evident and consistent at all levels of smoking, and even after adjusting for most important predictors of frailty. The proportion of current smokers is double in frail subjects in comparison to non-frail (14.8% vs. 7.2%, P < 0.0001). This ratio was confirmed by estimates of multivariate logistic regression analysis (OR = 1.29, 95%CI 1.22–1.34 for former smokers, and 2.34, 95%CI 2.17–2.53 for current smokers). The higher probability of being frail in smokers is present also in light smokers, even though the association becomes significant only in smokers of at least 16.9 pack-years (corresponding to the 5th decile) as shown in Fig. 6.

#### Diet

Univariate analyses showed a significant association between most dietary items investigated and frailty (Supplementary Table 3). However, major changes in recent diet due to illness make it difficult to attribute an etiological role to different dietary features. As a matter of fact, 33.8% of frail individuals declared to have had a major change in diet in the last 5 years due to illness, versus a percentage of 11.1% in non-frail subjects (P < 0.0001). As regards single dietary items, not a single food product showed an association with frailty strong enough to have clinical meaning, although in univariate analysis frailty was significantly associated with high intake (> 2 times per week) of processed meat, poultry, beef, lamb, and pork (P < 0.0001). On the opposite, never or occasional fish consumption was observed more frequently in frail subjects (45.8% never or less than once a week consumption of oily fish vs. 34.0% in non-frail, 35.3% vs. 28.4% for non-oily fish, both P < 0.0001). The consumption of dairy products was slightly higher in non-frail subjects, i.e., 92.2% milk consumers vs. 90.7% in frail, and 14.0% eating cheese 5–6 days per week or more vs. 9.5% in frail, (P < 0.0001). Vegetable and fruit consumption, as expected, was higher in non-frail subjects. The best protection is offered by a high consumption of raw vegetables, with a proportion of frail subjects 5% lower for every tablespoon/day (95%CI -3.6%–6.3%, P < 0.0001). No major difference in the consumption of vitamins was found in the study groups, while non-frail subjects had a higher consumption of minerals and other dietary supplements (OR = 0.733, 95%CI 0.689–0.779; P < 0.0001). After adjustment for major confounders, coffee or tea drinking were not any longer associated with the frailty status (data not shown).

#### Perinatal features

Two main parameters were included in the analysis, reflecting factors most investigated into the literature. Actually, both of them showed non-negligible associations with frailty status. Subjects receiving breastfeeding resulted in a lower probability of being frail (OR = 0.818; 95%CI 0.793–0.843; P < 0.0001), while this probability was increased for those whose mother smoked during pregnancy (OR = 1.249; 95%CI 1.182–1.319; P < 0.0001). Both estimates were adjusted for sex, age at the assessment of frailty, natural environment %, CRP (mg/L), smoking habit, and raw vegetable consumption.

#### Environmental conditions

ML results focused on several variables representing the environment where study subjects spend most of their life. The final model included the proportion of natural environment, NO_2_, NO_X_, PM10, and PM2.5. All these parameters were expressed as quantitative indexes which showed strong pairwise correlations among them [from a minimum of r = − 0.393 (natural environment %/PM10) to a maximum of r = 9.20 (NO_2_/NO_X_)]. Each parameter was evaluated separately to avoid distortions due to collinearity. The proportion of natural environment showed a negative association with frailty, with each additional percentage point in natural environment linked to a 0.8% lower probability of being frail. (95%CI 0.7–0.9; P < 0.0001). All the other markers of air pollution were positively associated with frailty, showing a higher probability of being frail in exposed subjects. Among them, the most predictive was PM2.5, with an OR = 1.241 for each additional µg/m^3^ of particulate (95%CI 1.212–1.27, P < 0.0001) (see Table 2).

**Table 2:** Binary logistic regression analysis of environmental quality and frailty status

Indexes of environmental quality	β	SE	P value	OR	95%CI
Natural environment %	− 0.008	0.001	0.000	0.992	0.991–0.993
Nitrogen dioxide air pollution (µg/m^3^) (NO_2_)	0.029	0.002	0.000	1.030	1.026–1.033
Nitrogen oxides air pollution (µg/m^3^) (NO_x_)	0.012	0.001	0.000	1.012	1.011–1.013
Particulate matter air pollution (µg/m^3^) (PM10)	0.053	0.007	0.000	1.054	1.040–1.069
Particulate matter air pollution (µg/m^3^) (PM2.5)	0.216	0.012	0.000	1.241	1.212–1.270

Estimates adjusted for sex, age at the assessment of frailty, C-reactive protein (mg/L), alcohol intake frequency, smoking status, breastfed as a baby, raw vegetable consumption

*β* regression coefficient, *SE* standard error, *OR* odds ratio, *95%CI* 95% confidence interval

#### Unhealthy workplace

Only 25.04% of subjects in the database provided information about the quality of the workplace where they have worked most of their lives. This low response rate diminished the influence of workplace quality in the overall analysis. Another factor limiting the contribution of workplace conditions to regression models was the high correlation between unhealthy conditions, e.g., 87% of subjects working in dusty environment worked also in noisy environment. The odds ratios reported in Table 3 show how essentially all unhealthy workplaces increase the risk of frailty, with increases ranging from 15.9 to 41.1%. Including more than one working condition in the same regression model generally resulted in a lower estimate of the OR.

**Table 3:** Binary logistic regression analysis of unhealthy conditions at workplace and frailty status

Working conditions (yes/no)	β	SE	P value	OR	95%CI
Workplace very noisy	0.206	0.080	0.010	1.229	1.050–1.438
Workplace very cold	0.344	0.076	0.000	1.411	1.216–1.636
Workplace very hot	0.246	0.078	0.002	1.279	1.097–1.490
Workplace very dusty	0.269	0.076	0.000	1.309	1.129–1.518
Workplace full of chemical or other fumes	0.169	0.083	0.042	1.184	1.006–1.393
Workplace with asbestos	0.358	0.110	0.001	1.430	1.152–1.774
Worked with paints, thinners or glues	0.148	0.096	0.122	1.159	.961–1.398
Worked with pesticides	0.321	0.162	0.048	1.378	1.002–1.894
Workplace had a lot of diesel exhaust	0.297	0.097	0.002	1.346	1.113–1.626

Estimates adjusted for sex, age at the assessment of frailty, C-reactive protein (mg/L), alcohol intake frequency, smoking status, breastfed as a baby, raw vegetable consumption

*β* regression coefficient, *SE* standard error, *OR* odds ratio, *95%CI* 95% confidence interval

#### Clinical features

The presence of polypharmacy is by far an alias of frailty, as confirmed by the OR associated with those subjects taking 5 or more different drugs, i.e. OR = 4.503; 95%CI 4.263–4.758; P < 0.0001). However, polypharmacy more than a predictor of frailty is a clinical sign of the syndrome, such as some of the biomarkers investigated. For a better understanding of the role of these biomarkers, all of them have been divided into quartiles, using for logistic regression analysis the lowest quartile as reference. A clear and dose-dependent association was found with the CRP, with higher probability of frailty in subjects in the highest quartile of CRP distribution (OR = 2.549; 95%CI 2.327–2.791; P < 0.0001). The relationship with vitamin D was inverse and highly significant, with an evident descending trend, reaching the OR of 0.457 in the 4th quartile (95%CI 0.422–0.494; P < 0.0001). Other biomarkers significantly associated with frailty status were the SHBG, the testosterone, and the IGF-1. As shown in Table 4, an increase of these parameters corresponds to a reduced probability of frailty. No association was found with oestradiol or rheumatoid factor.

**Table 4:** Binary logistic regression analysis of selected biomarkers used in clinics (quartiles) and frailty status

	β	SE	P value	OR	95%CI
CRP					
1st	–	–		1.000	–
2nd	0.247	0.049	0.0001	1.280	1.163–1.409
3rd	0.566	0.046	0.0001	1.761	1.610–1.927
4th	0.936	0.046	0.0001	2.549	2.327–2.791
Vitamin D					
1st	–			1.000	–
2nd	− 0.462	0.035	0.0001	0.630	0.588–0.675
3rd	− 0.756	0.039	0.0001	0.469	0.435–0.507
4th	− 0.784	0.040	0.0001	0.457	0.422–0.494
Oestradiol					
1st	–	–		1.000	–
2nd	0.161	0.139	n.s.	1.174	0.894–1.543
3rd	0.140	0.138	n.s.	1.150	0.877–1.549
4th	0.098	0.156	n.s.	1.103	0.812–1.498
Rheumatoid factor					
1st	–	–		1.000	–
2nd	− 0.125	0.114	n.s.	0.882	0.705–1.104
3rd	− 0.150	0.114	n.s.	0.861	0.688–1.077
4th	0.197	0.107	n.s.	1.218	0.988–1.501
SHBG					
1st	–	–		1.000	–
2nd	− 0.261	0.038	0.0001	0.770	0.715–0.830
3rd	− 0.373	0.039	0.0001	0.688	0.638–0.743
4th	− 0.490	0.040	0.0001	0.613	0.566–0.663
Testosterone					
1st	–	–		1.000	–
2nd	0.006	0.041	n.s.	1.006	0.928–1.090
3rd	− 0.650	0.075	0.0001	0.522	0.451–0.604
4th	− 1.090	0.078	0.0001	0.336	0.289–0.391
IGF-1					
1st	–	–		1.000	–
2nd	− 0.427	0.036	0.0001	0.653	0.608–0.700
3rd	− 0.552	0.038	0.0001	0.576	0.535–0.620
4th	− 0.506	0.037	0.0001	0.603	0.560–0.648

Estimates adjusted for sex, age at the assessment of frailty, C-reactive protein (mg/L), alcohol intake frequency, smoking status, breastfed as a baby, raw vegetable consumption

*β* regression coefficient, *SE* standard error, *OR* odds ratio, *95%CI* 95% confidence interval, *n.s.* not significant, *CRP* C-reactive protein, *SHBG* sex hormone binding globulin, *IGF-1* insulin-like growth factor

## Discussion

The present study aimed at investigating the possible association of frailty and pre-frailty in older adults with a wide spectrum of lifestyle, diet, environmental, occupational, and biomarker parameters in a large population of UK Biobank older adult participants, using traditional statistical and advanced data mining approaches. Our results confirm the multifactorial etiology of frailty, showing that the pathway to this condition begins early in life and is significantly influenced by factors such as lifestyle, smoking habits, diet, and working conditions. It remains challenging, however, to disentangle the interactions among these factors and understand how traits and state shape individual behaviour. To explore the relationship between potential predictors and frailty status in greater detail, the condition of pre-frailty was also considered. However, given the large number of conditions analyzed, a comprehensive discussion within a single paper was not feasible. These results did not reveal any distinctive patterns in relation to frailty and were therefore only presented for interested readers as univariate findings in the supplementary material.

The identification of subjects at higher risk of frailty represents a complex and multi-parametric process, requiring a detailed exploration of numerous parameters related to genetic, environmental, and behavioral factors. Advanced analytical techniques, such as PCA, are valuable tools for extracting meaningful insights from large datasets, revealing hidden relationships between variables and identifying the factors most strongly associated with frailty. This method facilitates the identification of a core set of conditions that can effectively predict frailty status. The choice to evaluate only variables showing a high degree of correlation with frailty reflects the need to optimize the number of parameters to be further considered for frailty prediction. The next step in the dimensionality reduction and recognition of features importance was played by machine learning techniques, which are effective in classifying frailty/non-frailty status, and exploring frailty-related factors (Jung et al. 2023; Szczepanowski et al. 2024). Jung and colleagues applied Min–Max scaling to normalize continuous/quantitative variables, and label encoding was used to convert categorical variables into numerical ones (Jung et al. 2023). Additionally, various balancing techniques were tested, with the SMOTE technique employed to balance the dataset. Top ranking factors were age and CRP, which ranked 1st and 2nd, respectively, among the 17 variables identified by PCA (Jung et al. 2023).

Based on the dataset identified by PCA and ML, a more conventional binary logistic regression analysis, in combination to evidence from univariate analysis, provided relevant information about main predictors of frailty. Not surprisingly, age was included in all final models as a key confounder. Ageing involves a progressive and general deterioration of the organism functions leading to a reduced capacity to react adaptively to changes and to preserve homeostasis (Félix et al. 2024). This general deterioration is faster in frailty, considered as “unsuccessful ageing”; therefore, increasing chronological age increases the likelihood of frailty (see Supplementary Table 3).

Interesting and partially unexpected results of our analysis concerned the lower risk of frailty in those subjects drinking daily or almost daily, and slightly lower in those drinking only occasionally, as compared to abstainers. Furthermore, the lower risk of being frail was also linked to occasional heavy drinking, but vanished for weekly heavy drinking, and reversed for daily heavy drinking. Results obtained in previous studies investigating the relationship between frailty and alcohol drinking often show an inverse association, both in cross-sectional (Woo et al. 2010, 2015; Romero-Ortuno 2014; Brinkman et al. 2018; DeClercq et al. 2020) and in longitudinal designs (Strandberg et al. 2018; Shah et al. 2018; Kojima et al. 2019a; Jazbar et al. 2021), and even in Mendelian randomization analysis (Lv et al. 2023; Xin et al. 2025). However, absence of linkage was also reported (Santiago et al. 2019; Gu et al. 2023; Guo et al. 2023). The U or J-shaped association found in our work between alcohol consumption and frailty, with moderate drinkers presenting the lowest frailty risk, has been described in earlier studies (Woods et al. 2005; Etman et al. 2015; Jazbar et al. 2021). The apparent protection against frailty offered by moderate alcohol use may be due to reverse causality or healthy survival effect among drinkers, since frailty itself may result in avoidance of alcohol consumption. In fact, our results also showed that frail subjects had a higher tendency to reduce drinking in the last ten years. Besides, drinking is often accompanied by social benefits, enhancing positive situations and facilitating socialization, thus preventing isolation that may contribute to frailty.

As regards the association between frailty and smoking, present results are consistent with previous observations. In cross-sectional studies, increased prevalence of frailty or higher frailty scores have been linked to smoking (Hubbard et al. 2009; Brinkman et al. 2018; DeClercq et al. 2020) and to exposure to secondhand tobacco smoke (García-Esquinas et al. 2015). Furthermore, highest levels of serum cotinine levels, indicative of recent exposure to tobacco smoke, were associated with higher odds of pre-frailty and frailty versus robustness in non-smoking (Fu et al. 2022) and smoking individuals (Xu et al. 2024). In longitudinal studies, baseline smoking status was observed as a strong predictor of frailty (Kojima et al. 2015, 2018a; Rodríguez-Laso et al. 2022). In addition, using a radically different approach, recent Mendelian randomization analyses indicated that smoking is associated with poor ageing phenotypes (Park et al. 2023) and with increased frailty (both frailty phenotype and frailty index) (Gu et al. 2023; Lv et al. 2023; Liu et al. 2023; Guo et al. 2023; Xin et al. 2025), supporting smoking as a causal risk factor of frailty. Physiologic changes associated with ageing and coexisting complex medical conditions may exacerbate the vulnerability of older adults to the effects of tobacco exposure (García-Esquinas et al. 2015). Nevertheless, mechanisms by which exposure to tobacco smoke contribute to frailty development are uncertain; they are likely to be diverse considering the numerous toxic chemicals contained in tobacco smoke. One of the most commonly suggested mechanisms is chronic inflammation, consistent with inflammageing as a driver of frailty, although epigenetic alterations, particularly smoking-induced DNA methylation, have been also suggested to play a role in smoking-associated development of frailty (Gao et al. 2017).

Regarding dietary parameters, our results showed that a high intake of processed meat, poultry, beef, lamb, and pork, and a low intake of fruits and vegetables, fish (oily and non-oily) and dairy products are linked to frailty, supporting results of previous studies. Thus, higher risk of frailty was found associated with a diet rich in red meat (either processed or unprocessed) (Struijk et al. 2022) or ultra-processed food (Sandoval-Insausti et al. 2020); oxidative stress was proposed to be potentially involved in this association (Van Hecke et al. 2017). Lower risk of frailty was reported as related to diets with high consumption of vegetables and fruits (Struijk et al. 2020; Fung et al. 2020; Sotos‐Prieto et al. 2022) and with Mediterranean diet, rich in those food products (Kojima et al. 2018b; Wang et al. 2018; Boucham et al. 2024). Frailty prevalence and incidence was also inversely associated with fish intake (Shibasaki et al. 2019; O’Connell et al. 2021; Maroto-Rodriguez et al. 2022; Ahn et al. 2023), and specifically with oily fish consumption (Del Brutto et al. 2020; Kim et al. 2024). The high-quality protein and branch chain amino acids found in fish were suggested as explanation for the relationship (O’Connell et al. 2021). Additionally, some studies have also shown that consumption of milk and/or certain dairy products in old age was effective in preventing frailty (Lana et al. 2015; Siefkas et al. 2023; Hong et al. 2024). A systematic review confirmed the importance of both quantitative (energy intake) and qualitative (nutrient quality) factors of nutrition in the development of frailty syndrome (Lorenzo-López et al. 2017). Interestingly, all these studies disclose modifiable dietary factors useful for interventions in frailty prevention.

A growing number of clinical trials have shown that CRP is related to many diseases, including cardiovascular disease, atherosclerotic vascular disease, systemic lupus erythematosus, cancer, and other diseases (reviewed in Zhou et al. 2024). CRP, which was strongly associated to frailty in all analyses, is an acute-phase protein that recognizes pathogens and host’s damaged cells to mediate their elimination by recruiting the complement system and phagocytic cells; it is a very useful non-specific biochemical marker of inflammation (Volanakis 2001). Chronic low-grade inflammation during ageing (inflammageing) has been pointed out as an underlying mechanism for frailty development (Marcos-Pérez et al. 2018). Indeed, elevated levels of inflammatory biomarkers, including CRP, have been reported to be linked to frailty in several meta-analyses (Mailliez et al. 2020; Marcos-Pérez et al. 2020a; Xu et al. 2022), agreeing with current results pointing at CRP as a major predictor of frailty status.

There is strong evidence supporting that long-term exposure to air pollution is associated with adverse health effects and mortality, even at levels below the USA National Ambient Air Quality Standards (Di et al. 2017). Specifically, a number of studies have suggested that exposure of older adults and susceptible individuals to air pollution is linked to reduction in life expectancy, poor lung function and respiratory mortality, and mental health impairment (Eckel et al. 2012; Shin et al. 2018; Zhang et al. 2019).

Air pollution is composed by a cocktail of substances (gases and particulate matter) originated mainly by combustion of petroleum or other hydrocarbon fuels by motor vehicles, and by natural or industrial processes that involve dust formation or gas releases. PM2.5, PM10, NO_2_ and NO_X_ are amongst the most frequently used indicators of air pollution. Positive associations have been previously reported between air pollution (outdoors and indoors) and frailty. Cao et al. showed that use of solid fuel (wood, coal, crop residue, and solid charcoal) for cooking, common in households from rural areas of China and indicative of poor indoor air quality, was positively associated with prevalence and incidence of phenotypic prefrailty and frailty as compared with use of clean cooking fuel (natural gas, marsh gas, liquefied petroleum gas and electric) (Cao et al. 2022). A cross-sectional study by Lee et al. revealed a significant association between PM2.5 exposure and frailty, with a stronger effect in vulnerable groups (Lee et al. 2020). Similarly, Shin et al. reported associations between more than a year of exposure to PM2.5 and PM10 and frailty, with slighter effects in the pre-frail group (Shin and Choi 2021). A very recent study by Veronese et al., conducted in UK Biobank participants using ordinal logistic regression, showed that the increment in the exposure to PM2.5-10 and to NO_X_ was associated with a higher probability of being both pre-frail and frail (Veronese et al. 2023). In longitudinal studies, chronic exposure to PM2.5 was reported to be related to increased odds of developing post-myocardial infarction frailty (Myers et al. 2013), and long-term exposure to air pollution was evidenced to contribute to higher incidence of frailty, additionally suggesting that worsening air quality may influence poorer frailty trajectories (Hu et al. 2020). In addition, studying frailty transitions, Guo et al. observed that long-term exposure to PM was associated with higher risks of worsening and lower risks of improvement in frailty (Guo et al. 2024). Similarly, using Mendelian randomization, Xiao et al. found a significant causal association between PM2.5 and frailty (Xiao et al. 2023). In the current study, the percentage of natural environment was strongly correlated with the level of PM2.5, PM10, and those of NO_x_ and NO_2_, where lower concentrations indicate higher air quality. In our analysis, we observed the substantial influence of all parameters of air quality on the prediction of frailty/non-frailty conditions, confirming all previously reported associations between air pollution and frailty. From our results, the most predictive environmental parameter for frailty was the PM2.5, agreeing with the largest general concern related to this indicator, since these particles may descend more deeply into the respiratory tract due to the lower particle size, and they likely contain a more hazardous mixture of substances (Rajagopalan et al. 2018). Furthermore, PM2.5 is now recognized as an accelerator of human ageing and a predisposing factor for several age-related diseases (Wang et al. 2024).

Regarding the possible molecular mechanisms by which exposure to air pollution increases frailty risk, accumulated evidence suggests that air pollution, along with consistent exposure to PM2.5, induces inflammation, among other effects (Delfino et al. 2009; Hart et al. 2013; Rückerl et al. 2014). As mentioned above, inflammageing is suspected to be involved in frailty development, and it is mediated by elevated levels of proinflammatory molecules, including CRP. Hence, the dose-dependent association between CRP and frailty found in the current study and in several previous meta-analyses (Mailliez et al. 2020; Marcos-Pérez et al. 2020a; Xu et al. 2022) might mediate the relationship between chronic exposure to air pollution and frailty.

Similarly, vitamin D is crucial for the maintenance of immune system homeostasis (reviewed in Daryabor et al. 2023), and its deficiency is involved in a variety of chronic diseases, including renal, cardiovascular, bone, metabolic and muscular disorders, among others (reviewed in Paul et al. 2024). Some meta-analyses demonstrated that vitamin D deficiency is also associated with the risk of frailty in older adults (Zhou et al. 2016; Ju et al. 2018; Marcos-Pérez et al. 2020b); therefore, it is not surprising that our study found an inverse and highly significant relationship between vitamin D and frailty. Lower concentrations of vitamin D, together with higher concentrations of CRP were also found to be linked to frailty in UK Biobank participants using a multiple linear regression approach (Chu et al. 2023). Additional associations with frailty found in that study (Chu et al. 2023) were consistent with our results, such as for lower levels of IGF-1 (circulating hormone with anabolic effects on muscle whose dysregulation has been implicated in reduction in muscle mass and strength, cardiovascular disease, diabetes and cancer) (reviewed in Yang et al. 2005), and higher concentrations of SHBG (serum glycoprotein involved in hyper- and hypoandrogenism, thyroid disorders, pituitary diseases, liver disorders, and breast as well as prostate cancer) (reviewed in Thaler et al. 2015).

Polypharmacy may increase the risk for inappropriate drug use. Specific tests to detect the majority of age-related pharmacological changes are usually not available in everyday clinical practice, which limits the estimation of drug risks and possibilities to individualize drug therapy in geriatric patients before drug prescription (Fialová et al. 2019). Furthermore, age-related pharmacokinetic and pharmacodynamic modifications in response to drugs can be exacerbated in frail subjects (Shi et al. 2008), increasing their susceptibility to adverse drug reactions. Indeed, there appears to be a relationship between frailty and medication harm (reviewed by Lam et al. 2022). Although a systematic review by Gutiérrez-Valencia et al. suggested that the causal relationship between polypharmacy and frailty is unclear and appears to be bidirectional, results from the current and the abovementioned studies are consistent with the hypothesis that overprescribing may contribute to frailty in older adults (Gutiérrez‐Valencia et al. 2018). In a recent longitudinal study, polypharmacy was associated with frailty worsening within a relatively short period, increasing the risk of transitioning from the robust state to frailty (Lorenzo-López et al. 2019). These findings highlight the importance of closely monitoring polypharmacy in older adults to optimize health outcomes.

The large variety of endogenous and exogenous factors associated to frailty in this analysis, recall the concept of exposome, a study model that applies when the outcome is influenced by several exposures, from conception onward, over a complete lifetime (Vineis et al. 2020). Environmental factors considered in the exposome contribute to the acceleration of ageing processes, and therefore should be considered by epidemiological studies, according to the idea of accumulated biological capital of individuals (Vineis and Barouki 2022; Pandics et al. 2023). Under this perspective, the increased risk of frailty associated with unhealthy workplaces supported the idea that occupational exposures during adulthood may contribute to frailty development in later life.

Among the most interesting results is the evidence that major contributors to the exposome in early life, such as maternal smoking around birth and breastfed as a baby, are associated with the frailty condition in older age. There is strong evidence for an association between smoking during pregnancy and low birth weight (Suzuki et al. 2008; Zheng et al. 2016). Low birth weight was reported as associated with frailty in older age in the Helsinki Birth Cohort Study (Haapanen et al. 2018), and in a Mendelian randomization analysis (Gu et al. 2023), supporting the hypothesis that the risk of frailty can be influenced by the interrelationship between maternal smoking during pregnancy and low birth weight.

Two former studies conducted in UK Biobank participants without age restrictions found that non-breastfed was associated with accelerated biological ageing (including frailty phenotype) (Gao et al. 2024) and with a higher frailty index (Maharani et al. 2023) in the late-life; our findings in participants aged 60+ using advanced data mining methods reinforce those previous results.

The present study has a number of strengths, starting from the large sample size used for the analysis; data from UK Biobank offered the opportunity to manage a huge number of data (from more than 200,000 individuals aged 60 years and over), thus providing the analysis with a strong robustness and reliability, additionally strengthened by the advanced data mining techniques employed, including PCA and ML. Furthermore, participants of the UK Biobank are community-dwelling individuals (Sudlow et al. 2015), who are not institutionalized, assuring that frailty is not overrepresented in the sample. Among the study limitations, we must firstly mention that results obtained for associations with frailty phenotype may not be necessarily applicable to frailty as determined by other different tools, including the widely used Frailty Index. In fact, although moderate correspondence has been reported for these two scales, some authors consider that they are not equivalent but complementary (Cesari et al. 2014). Secondly, the cross-sectional design of the study did not allow us to assess the temporal association between frailty and the different factors investigated. A third limitation is determined by the age of the study participants, since data from individuals older than 82 (who are likely to present a higher prevalence of frailty) were not available from the UK Biobank, thus restricting the generalizability of the findings to the oldest old population sector (< 85 years or centenarians). And lastly, comorbidity was not accounted for in the analyses, but since one of the major reasons for polypharmacy is the presence of multiple disease states (Shi et al. 2008), it was indirectly considered with the polypharmacy.

In conclusion, our study provided robust and original evidence on the association between a large battery of potential risk factors, from early to later stages of life, and the occurrence of frailty in older age, thus advancing our knowledge of the lifestyle and environmental drivers of frailty. Despite their complexity, these results will contribute to the development of prevention strategies and facilitate the early detection of individuals at high risk of developing frailty. A deeper insight into risk factors associated to pre-frailty might contribute to anticipate interventions on most effective risk factors.

## Supplementary Information

Below is the link to the electronic supplementary material.

Supplementary file1 (DOCX 398 KB)

## Data Availability

The UK Biobank data are available via direct application (www.ukbiobank.ac.uk/register-apply).
