# Supplementary material for: Lifestyle, environment and other major determinants of frailty in older adults: a population-based study from the UK Biobank
Source: Biogerontology. 2025 May 3;26(3):100. doi: 10.1007/s10522-025-10242-x (PMC12049404; doi:10.1007/s10522-025-10242-x)

**Supplementary information**

**Lifestyle, environment and other major determinants of frailty in older adults. A population-based study from the UK Biobank**

**Geroscience**

Ali Hemadeh · Carlota Lema-Arranz · Stefano Bonassi · Leonardo Buscarini · Francesco Infarinato · Paola Romano · Alessia Finti · Franco Marinozzi · Fabiano Bini · Natalia Fernández-Bertólez · João Paulo Teixeira · Laura Lorenzo-López · Vanessa Valdiglesias · Blanca Laffon

Corresponding author: L. Lorenzo-López. Universidade da Coruña, Gerontology and Geriatrics Research Group, Instituto de Investigación Biomédica de A Coruña (INIBIC), Complexo Hospitalario Universitario de A Coruña (CHUAC), Sergas, A Coruña, Spain

**Supplementary Material 1**

Computation of correlation coefficients by parametric angular range between vectors representing variables.

The coefficients in the PCA output represent the loadings of each variable in defining each principal component. They were used to calculate the angle between two vectors representing the loadings assumed by the variables along the PC to assess their correlation with the variable frailty status (FRAILTY_3cat_def) through the cosine of the angle. This process was carried out in relation to the biplot graph, in which the coefficient of a given variable corresponding to the first PC is representative of the abscissa of a point on the graph, and the coefficient of the second PC is representative of the ordinate. The vector of each variable was compared to the frailty vector by measuring the angle formed between the two. The angle between the frailty vector and each variable's vector was determined by calculating the two-dimensional arctangent between them. The cosine of these angles was subsequently studied to assess their correlation [1],[2]. As observable in Fig. 1 below, a parametric angular range was set around the frailty status. Specifically, variables within a range of ±10°, ±20°, ±30° were considered. These angular ranges were converted to absolute values for cosine calculations, as the primary interest was the general proximity of other vectors to the frailty vector. The ranges of ±10°, ±20°, ±30° represent, in terms of the absolute value of the cosine of the angle, a correlation index of at least 0.98 for the first range, 0.94 and 0.86 for the subsequent ones.


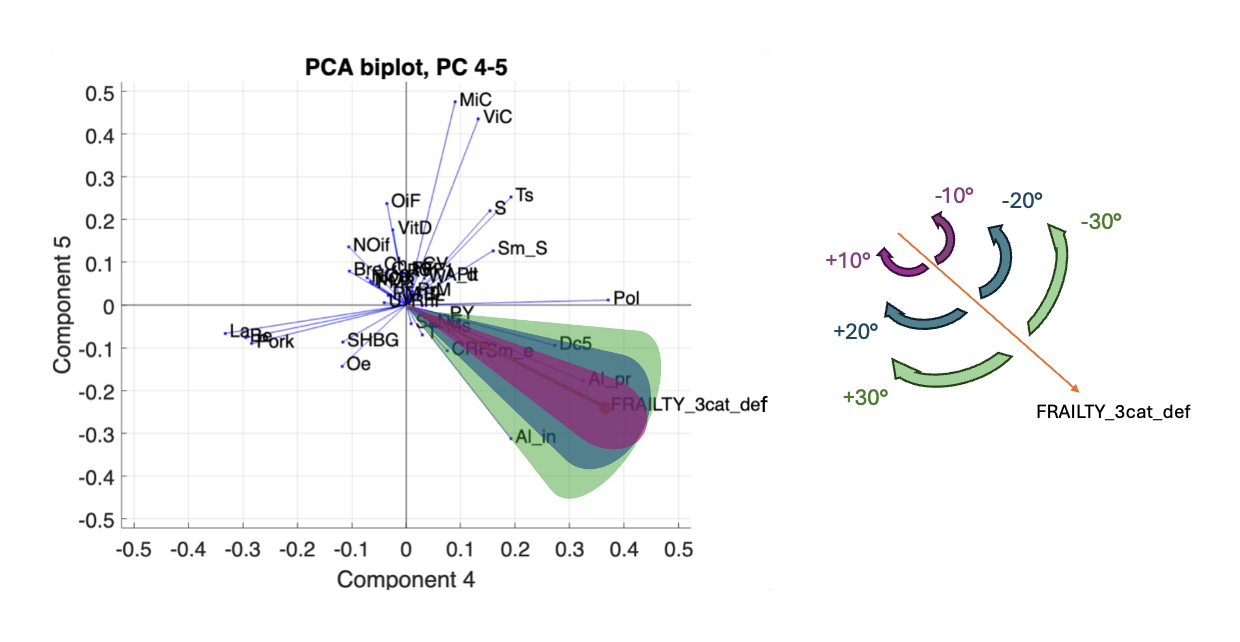


**Fig*.* 1**. Representation of the parametric angular range, centered on the frailty status (FRAILTY_3cat_def), used for correlation coefficient computations. The parametric angular range emphasizes the proximity of other vectors to the frailty vector, FRAILTY_3cat_def. The ±10° range corresponds to a correlation index of at least 0.98, while the subsequent ±20° and ±30° ranges show correlation indices of 0.94 and 0.86, respectively. See Supplementary Table 1 for notation and acronym explanations.

**Supplementary Table 1** Notations and acronyms for the parameters used in the analyses (see Methods, section Lifestyle, diet, and other parameters included in the statistical analysis for additional details). The table includes also variables that did not survived the PCA screening and are not reported in the graphs

| Parameter notation | Acronym (used in biplot graphs) | Parameter |
| --- | --- | --- |
|  |  |  |
| FRAILTY_3cat_def | FRAILTY_3cat_def | Frailty status |
| Sex | S | Sex |
| Age_def | A_d | Age at the assessment of frailty |
| Alcohol_status |  | Alcohol drinker status |
| Alcohol_intake_freq | Al_in | Alcohol intake frequency |
| Alcohol_6U |  | Frequency of consuming 6 or more units of alcohol |
| Alcohol_previous | Al_pr | Alcohol intake compared to 10 years ago |
| Smoking_status | Sm_S | Smoking status |
| Pack_years | PY | Pack-years |
| Smoke_exposure_total | Sm_e | Exposure to tobacco smoke |
| Cooked_vegetables | CV | Cooked vegetable intake |
| Raw_vegetables | RV | Raw vegetable intake |
| Fresh_fruit | FF | Fresh fruit intake |
| Oily_fish | OiF | Oily fish intake |
| Non_Oily_fish | NOif | Non-oily fish intake |
| Processed_meat | PrM | Processed meat intake |
| Poultry | Plt | Poultry intake |
| Beef | Be | Beef intake |
| Lamb | La | Lamb intake |
| Pork | Pork | Pork intake |
| Cheese | Ch | Cheese intake |
| Milk | Sa | Milk intake |
| Bread | B | Bread intake |
| Cereal | C | Cereal intake |
| Salt |  | Salt added to food |
| Tea | T | Tea intake |
| Coffee |  | Coffee intake |
| Water | W | Water intake |
| Diet_changes_5y | Dc5 | Major dietary changes in the last 5 years |
| Vitamins_cat | ViC | Vitamin and mineral supplements |
| Minerals_cat | MiC | Mineral and other dietary supplements |
| Breastfed | Bre | Breastfed as a baby |
| Maternal_smoking | Ms | Maternal smoking around birth |
| Workplace_noisy |  | Workplace very noisy |
| Workplace_cold |  | Workplace very cold |
| Workplace_hot |  | Workplace very hot |
| Workplace_dusty |  | Workplace very dusty |
| Workplace_chemicals |  | Workplace full of chemical or other fumes |
| Workplace_asbestos |  | Workplace with materials containing asbestos |
| Workplace_paints |  | Worked with paints, thinners or glues |
| Workplace_pesticides |  | Worked with pesticides |
| Workplace_diesel |  | Workplace had a lot of diesel exhaust |
| Natural_env_percent | NE | Natural environment % |
| Polypharmacy | Pol | Polypharmacy |
| NO2 | NO2 | Nitrogen dioxide air pollution (NO_2_) |
| NOX | NOX | Nitrogen oxides air pollution (NO_X_) |
| PM10 | PM10 | Particulate matter air pollution (PM10) |
| PM2 | PM2 | Particulate matter air pollution (PM2.5) |
| UV_protection | UV | Use of sun/UV protection |
| Freq_solarium_cat |  | Frequency of solarium/sunlamp use |
| CRP | CRP | C-reactive protein (CRP) |
| IGF1 | IGF1 | Insulin-like growth factor 1 (IGF-1) |
| Oestradiol | Oe | Oestradiol |
| Rheumatoid_factor | RhF | Rheumatoid factor |
| SHBG | SHBG | Sex hormone binding globulin (SHBG) |
| Testosterone | Ts | Testosterone |
| VitD | VitD | Vitamin D |

**Supplementary Table 2** Loading values of variables from the first 10 principal components (PC) generated by the principal component analysis (PCA). Each loading represents the contribution of a variable to a specific component. For notation and acronym explanations refer to Supplementary Table 1

| **ACRONYM** | **PC 1** | **PC 2** | **PC 3** | **PC 4** | **PC 5** | **PC 6** | **PC 7** | **PC 8** | **PC 9** | **PC 10** |
| --- | --- | --- | --- | --- | --- | --- | --- | --- | --- | --- |
| **FRAILTY_3**  **Cat_def** | -0.001474343 | 0.095807122 | 0.241935968 | 0.367101185 | -0.237805517 | -0.034105984 | 0.031293327 | 0.013859326 | 0.102345705 | 0.000904585 |
| **A_d** | 0.022093462 | 0.007433492 | 0.063648763 | 0.061756022 | 0.066255604 | -0.02459775 | 0.230152414 | 0.117261852 | 0.605768723 | -0.046587522 |
| **PY** | 0.096459174 | 0.039442207 | 0.00357788 | 0.071940643 | -0.0238705 | 0.005852569 | 0.004046814 | 0.022495386 | -0.047492532 | 0.218223621 |
| **CV** | -0.031617262 | 0.002921151 | 0.102858468 | 0.021550068 | 0.091188816 | 0.287929315 | -0.024238709 | -0.17304742 | 0.014954178 | 0.160488556 |
| **RV** | -0.099676599 | 0.010738642 | 0.065674101 | -0.007018123 | 0.074892715 | 0.301374412 | -0.096151856 | -0.037311308 | 0.046093629 | 0.218764111 |
| **FF** | -0.200812669 | 0.013134316 | 0.066656868 | 0.001597271 | 0.079878382 | 0.249927677 | 0.119252307 | -0.07625747 | -0.004411322 | 0.0941599 |
| **B** | 0.085826837 | -0.011104996 | -0.01630725 | 0.024446576 | 0.020580082 | -0.010858757 | 0.182427165 | 0.315593664 | -0.053940973 | 0.122055168 |
| **C** | -0.069092981 | -0.031955824 | -0.076778484 | -0.035563358 | 0.079500635 | -0.050066045 | 0.494703419 | -0.101776099 | 0.062756022 | -0.118919188 |
| **T** | -0.007351041 | -0.00350478 | 0.032785787 | 0.029440531 | -0.069673308 | 0.011242807 | 0.175110014 | 0.31521597 | -0.212863713 | -0.037737559 |
| **W** | -0.14276495 | 0.033315429 | 0.113844788 | 0.032826385 | 0.061788446 | 0.195570785 | -0.120132375 | -0.146567776 | 0.051303497 | 0.142288816 |
| **NE** | -0.025217024 | -0.365994404 | 0.030895044 | 0.048446791 | -0.04423623 | -0.003667786 | 0.003264577 | 0.017958373 | -0.009844137 | -0.026048184 |
| **NO2** | 0.039402624 | 0.518975579 | -0.04543226 | -0.071327158 | 0.063813748 | 0.004619026 | 0.027742894 | -0.001537391 | -0.006701932 | -0.031995451 |
| **NOX** | 0.04013386 | 0.51379028 | -0.041836352 | -0.06613567 | 0.054993586 | 0.004660481 | 0.034077653 | 0.013844093 | -0.013901357 | -0.044364534 |
| **PM10** | 0.017821199 | 0.262497006 | -0.019427868 | -0.033655036 | 0.022460637 | -0.016056018 | -0.016875223 | -0.023979945 | 0.008827229 | 0.069288243 |
| **PM2** | 0.037672425 | 0.46629508 | -0.031703148 | -0.062512845 | 0.050175707 | -0.000664595 | 0.027019562 | 0.012522292 | -0.008353109 | -0.032475391 |
| **CRP** | 0.010997607 | 0.024633603 | 0.070135089 | 0.074911796 | -0.10712479 | -0.049633807 | -0.094828383 | 0.036352676 | 0.096319394 | 0.19585253 |
| **IGF1** | 0.02508388 | -0.016687134 | -0.044836157 | 0.005376698 | 0.076709124 | 0.060592489 | 0.169269957 | -0.060580192 | -0.298432014 | -0.041456753 |
| **Oe** | -0.213151917 | 0.02816008 | 0.016688921 | -0.118090077 | -0.143326862 | -0.072877261 | -0.166081854 | 0.069342078 | 0.042088795 | -0.103419773 |
| **RhF** | 0.001692352 | 0.014759595 | 0.00627578 | 0.000297125 | 0.005405467 | -0.001784319 | -0.009510643 | 0.04708544 | 0.110603075 | -0.076062451 |
| **SHBG** | -0.154561794 | 0.009818242 | -0.00855092 | -0.116748951 | -0.086786353 | -0.081336434 | -0.08980429 | 0.235269073 | 0.203302151 | -0.19522415 |
| **Ts** | 0.383244877 | -0.04144472 | -0.02885538 | 0.192359854 | 0.252891585 | 0.072216492 | 0.145750858 | -0.10495708 | -0.062281587 | 0.021079387 |
| **VitD** | -0.042493763 | -0.037898544 | 0.021783599 | -0.024109136 | 0.175548925 | -0.088937965 | 0.030708789 | 0.006316201 | -0.08836429 | -0.236982205 |
| **S** | 0.493716932 | -0.050840547 | -0.059891234 | 0.153266495 | 0.220984435 | 0.075861028 | 0.083519514 | -0.04190055 | -0.017020278 | -0.021526207 |
| **Al_s** | -1.57772E-30 | -7.88861E-31 | 3.23117E-27 | -5.16988E-26 | -4.1359E-25 | 4.23516E-22 | 0 | -2.77556E-17 | 6.93889E-18 | 0 |
| **Al_in** | -0.200914177 | 0.071521132 | 0.063222474 | 0.192014696 | -0.313165509 | -0.042353825 | 0.341528986 | 0.124671599 | -0.121443557 | 0.182963067 |
| **Al_6U** | 3.08149E-33 | -7.70372E-34 | -5.04871E-29 | 2.01948E-28 | 0 | -6.61744E-24 | 1.69407E-21 | 3.46945E-18 | 0 | 8.32667E-17 |
| **Al_pr** | 0.000376375 | 0.073832106 | 0.169772803 | 0.325267961 | -0.177884897 | 0.095217514 | 0.089134433 | -0.033669693 | -0.090406763 | -0.062001517 |
| **Sm_S** | 0.158241937 | 0.012637053 | 0.078854886 | 0.159223343 | 0.12739008 | 0.060978745 | -0.301987125 | -0.212003479 | 0.236037624 | -0.211941722 |
| **Sm_e** | 0.121790715 | 0.075001234 | 0.139921913 | 0.137773629 | -0.111922121 | -0.055307756 | -0.226354841 | 0.17532458 | -0.129070335 | 0.066849051 |
| **OiF** | -0.129587706 | -0.007453581 | 0.128565149 | -0.035331668 | 0.23801751 | 0.438826262 | 0.041373223 | 0.192571048 | 0.08366632 | -0.131972591 |
| **NOif** | -0.025963202 | -0.023057925 | 0.153227435 | -0.105108933 | 0.135897145 | 0.321965309 | 0.02418251 | 0.42112317 | 0.07937519 | -0.209340772 |
| **PrM** | 0.282395497 | -0.015359108 | 0.130887493 | 0.011993561 | 0.031162609 | -0.004665173 | -0.075201776 | 0.383758847 | -0.051520363 | 0.055748688 |
| **Plt** | -0.055236364 | 0.010843435 | 0.036936284 | 0.084219249 | 0.066860842 | 0.239465821 | -0.090617588 | 0.086620956 | -0.212600818 | 0.047140009 |
| **Be** | 0.156241148 | -0.019384785 | 0.384257935 | -0.294288279 | -0.076885744 | -0.065708637 | 0.078602553 | -0.08473421 | -0.046091132 | -0.017855024 |
| **La** | 0.14906064 | -0.0070232 | 0.461464923 | -0.332423077 | -0.066505356 | -0.038740488 | 0.076810821 | -0.136162147 | -0.026111094 | 0.01180009 |
| **Pork** | 0.141576141 | -0.011657086 | 0.427950695 | -0.283319755 | -0.090322805 | -0.026299344 | 0.09248047 | -0.083277047 | -0.049110935 | 0.03764975 |
| **Ch** | 0.041084795 | -0.021367578 | -0.040296672 | -0.048430131 | 0.091383935 | 0.037316477 | -0.084774342 | 0.293316245 | -0.061973282 | 0.29206786 |
| **Mi** | -3.50325E-46 | -8.75812E-47 | 0 | -2.29589E-41 | 0 | 0 | 0 | -3.30872E-24 | 0 | 5.42101E-20 |
| **Sa** | 0.069677475 | 0.012753582 | 0.062003322 | 0.00945243 | -0.043631411 | -0.113702422 | -0.349614689 | 0.127506424 | 0.142508129 | 0.093612745 |
| **Co** | 0 | 1.75162E-46 | -2.86986E-42 | -4.59177E-41 | 7.34684E-40 | 0 | 0 | 0 | 0 | 0 |
| **Dc5** | -0.111451612 | 0.052332887 | 0.165602171 | 0.272796299 | -0.095023257 | 0.172338014 | -0.042609178 | -0.142998737 | -0.114390421 | -0.115049714 |
| **ViC** | -0.234934858 | 0.015354267 | 0.217698529 | 0.132398562 | 0.435352738 | -0.346652682 | -0.042207705 | 0.037427539 | -0.122231499 | 0.078682458 |
| **MiC** | -0.243026235 | -0.007522773 | 0.200312732 | 0.089400445 | 0.475472142 | -0.33708428 | 0.002571136 | 0.042430703 | -0.080518577 | 0.042755008 |
| **Bre** | -0.103362788 | 0.002740003 | 0.000429876 | -0.104021411 | 0.07855599 | 0.075416744 | -0.087759704 | -0.086224911 | -0.04114576 | 0.419507567 |
| **Ms** | 0.037377895 | 0.014490789 | 0.036142599 | 0.065915691 | -0.05365397 | 0.003256791 | -0.214319086 | 0.031723322 | -0.371978231 | -0.401623336 |
| **WN** | 0 | 0 | 0 | 0 | 0 | 0 | 0 | 0 | 0 | 0 |
| **WC** | 0 | 0 | 0 | 0 | 0 | 0 | 0 | 0 | 0 | 0 |
| **WH** | 0 | 0 | 0 | 0 | 0 | 0 | 0 | 0 | 0 | 0 |
| **WD** | 0 | 0 | 0 | 0 | 0 | 0 | 0 | 0 | 0 | 0 |
| **WC** | 0 | 0 | 0 | 0 | 0 | 0 | 0 | 0 | 0 | 0 |
| **WAS** | 0 | 0 | 0 | 0 | 0 | 0 | 0 | 0 | 0 | 0 |
| **WPa** | 0 | 0 | 0 | 0 | 0 | 0 | 0 | 0 | 0 | 0 |
| **WPe** | 0 | 0 | 0 | 0 | 0 | 0 | 0 | 0 | 0 | 0 |
| **Wdi** | 0 | 0 | 0 | 0 | 0 | 0 | 0 | 0 | 0 | 0 |
| **Pol** | -0.00860225 | 0.059249748 | 0.297819663 | 0.371115989 | 0.011390752 | -0.086792212 | 0.053054983 | 0.012127118 | 0.174896741 | -0.016645745 |
| **UV** | -0.213921909 | 0.008978978 | 0.090698213 | -0.040022171 | 0.005368995 | 0.12229298 | -0.009403558 | -0.086144924 | -0.077753862 | -0.218429858 |
| **FreS** | 0 | 0 | 0 | 0 | 0 | 0 | 0 | 0 | 0 | 0 |

**Supplementary Table 3** Univariate analysis of the association between relevant variables included in the UK biobank and frailty status. Continuous variables are reported as mean±standard deviation, categorical variables are reported as number (%)

| **Variables** | **All**  **N=221.896** | **Non-Frail**  **N=119.332** | **Pre-Frail**  **N= 93.180** | **Frail**  **N= 9384** | **P value** |
| --- | --- | --- | --- | --- | --- |
| Age (years) | 64.8±3.6 | 64.6±3.5 | 65.02±3.7 | 64.9±3.6 | *<0.001* |
| Sex  *Male*  *Female* | 105272(47.4%)  116624(52.6%) | 57439(48.1%)  61893(51.9%) | 43642(46.8%)  49538(53. 2%) | 4191(44.7%)  5193(55.3%) | *<0.001* |
| Natural environment (%) | 42.7±25.6 | 44.2±25.9 | 41.4±25.2 | 36.9±23.8 | *<0.001* |
| UV protection  *Never/rarely*  *Sometimes*  *Most of the time*  *Always*  *Do not go out in sunshine* | 22208(10.0%)  75528(34.0%)  76413(34.4%)  46275(20.9%)  1332(0.6%) | 10024(8.4%)  41056(34.4%)  42983(36.0%)  24845(20.8%)  395(0.3%) | 10446(11.2%)  31335(33.6%)  31091(20.8%)  19510(20.9%)  712(0.8%) | 1738(18.5%)  3137(33.4%)  2339(24.9%)  1920(20.5%)  225(2.4%) | *<0.001* |
| Nitrogen dioxide air pollution (µg/m^3^) | 26.1±7.4 | 25.5±7.26 | 26.5±7.4 | 28.1±7.6 | *<0.001* |
| Nitrogen oxides air pollution (µg/m^3^) | 42.97±15.03 | 42.02±14.63 | 43.77±15.28 | 47.09±16.35 | *<0.001* |
| Particulate matter air pollution (PM10) (µg/m^3^) | 16.15±1.89 | 16.08±1.90 | 16.2±1.80 | 16.4±1.80 | *<0.001* |
| Particulate matter air pollution (PM2.5) (µg/m^3^) | 9.9±1.03 | 9.8±1.01 | 9.97±1.03 | 10.2±1.09 | *<0.001* |
| CRP (mg/L) | 2.7±4.6 | 2.3±4.06 | 3.0±4.8 | 4.96±7.2 | *<0.001* |
| IGF-1 (nmol/L) | 20.33±5.4 | 20.6±5.2 | 20.1±5.6 | 19.04±6.2 | *<0.001* |
| Oestradiol (pmol/L) | 284.1±223.2 | 289.9±237.9 | 277.2±204.0 | 281.0±217.8 | *<0.001* |
| Rheumatoid factor (IU/mL) | 24.87±20.03 | 24.23±19.22 | 25.2±20.3 | 28.92±25.0 | *<0.001* |
| SHBG (nmol/L) | 52.2±25.5 | 52.7±25.2 | 51.8±25.6 | 49.96±27.2 | *<0.001* |
| Testosterone (nmol/L) | 6.8±6.02 | 6.9±6.7 | 6.7±5.97 | 6.07±5.6 | *<0.001* |
| Vitamin D (nmol/L) | 50.88±20.8 | 52.42±20.5 | 49.7±20.81 | 42.7±21.4 | *<0.001* |
| Smoking status  *Prefer not to answer*  *Never*  *Previous*  *Current* | 925(0.4%)  113.011(50.9%)  91.264(41.1%)  16696(7.5%) | 411(0.3%)  62738(52.6%)  48530(40.8%)  7653(6.4%) | 422(0.5%)  46372(49.8%)  38719(41.6%)  7667(8.3%) | 92(1%)  3901(41.6%)  4015(42.8%)  1376(14.7%) | *<0.001* |
| Pack-years | 25.9±20.5 | 23.3±18.4 | 27.5±21.4 | 36.1±26.1 | *<0.001* |
| Exposure to tobacco smoke (smokers+2nd hand)  *No*  *Yes* | 150592 (74.3%)  51970 (25.7%) | 84865(77.2%)  25068 (22.8%) | 60814(72.0%)  23617(28.0%) | 4913(59.9%)  3285(40.1%) | *<0.001* |
| Alcohol drinker status  *Never*  *Previous*  *Current* | 10089 (4.5%)  8401(3.8%)  203255 (91.7%) | 4188(3.5%)  3137(2.6%)  111969(93.9%) | 4988(5.4%)  4231(4.5%)  83873(90.1%) | 913(9.8%)  1033(11.0%)  7413(79.2%) | *<0.001* |
| Alcohol intake frequency  *Daily or almost daily*  *3-4 times a week*  *1-2 a week*  *1-3 times a month*  *Special occasions only*  *Never* | 50218(22.6%)  50791(22.9%)  53622(24.2%)  22155(10%)  26469(11.9%)  18535(8.4%) | 30007(25.2%)  30174(25.3%)  28974(24.3%)  11271(9.4%)  11543(9.7%)  7334(6.1%) | 19038(20.4%)  19459(20.9%)  22688(24.4%)  9858(10.6%)  12830(13.8%)  9247(9.9%) | 1173(12.5%)  1158(12.4%)  1960(20.9%)  1026(11.0%)  2096(22.4%)  1954(20.9%) | *<0.001* |
| Alcohol frequency >6U  *Never*  *Less than monthly*  *Monthly*  *Weekly*  *Daily or almost daily* | 38597(59.3%)  13472(20.7%)  4589(7.0%)  6387(9.8%)  2096(3.2%) | 22604(58.5%)  8142(21.1%)  2837(7.3%)  3835(9.9%)  1196(3.1%) | 15141(60.0%)  5134(20.3%)  1685(6.7%)  2445(9.7%)  842(3.3%) | 852(66.6%)  196(15.3%)  67(5.2%)  107(8.4%)  58(4.5%) | *<0.001* |
| Alcohol intake vs. 10 years ago  *No answer*  *More nowadays*  *About the same*  *Less nowadays* | 1163(0.6%)  28616(14.1%)  82355(40.5%)  91121(44.8%) | 513(0.4%)  17262(15.4%)  49441(44.2%)  44753(40.0%) | 561(0.7%)  10675(12.7%)  30994(37.0%)  41643(49.7%) | 89(1.2%)  679(9.2%)  1920(25.9%)  4725(63.7%) | *<0.001* |
| Cooked vegetable intake (tablespoons/day) | 2.7±2.4 | 2.8±2.2 | 2.7±2.6 | 2.3±3.1 | *<0.001* |
| Raw vegetable intake (tablespoons/day) | 1.7±3.3 | 1.7±3.2 | 1.6±3.3 | 1.2±3.6 | *<0.001* |
| Oily fish intake  *No answer*  *Never*  *Less than once a week*  *Once a week*  *2-4 times a week*  *5-6 times a week*  *Once or more daily* | 856 (0.4%)  17416 (7.8%)  62291 (28.1%)  91674 (41.3%)  47297 (21.3%)  1805 (0.8%)  557 (0.3%) | 289 ( 0.2%)  7749 (6.5%)  32838 (27.5%)  51213 (42.9%)  26033 (21.8%)  959 (0.8%)  251 ( 0.2%) | 452 (0.5%)  8248 (8.9%)  26569 (28.5%)  37194 (39.9%)  19675 (21.1%)  772 (0.8%)  270 (0.3%) | 115 (1.2%)  1419 (15.1%)  2884 (30.7%)  3267 (34.8%)  1589 (16.9%)  74 (0.8%)  36 (0.4%) | *<0.001* |
| Non-oily fish intake  *No answer*  *Never*  *Less than once a week*  *Once a week*  *2-4 times a week*  *5-6 times a week*  *Once or more daily* | 753(0.3%)  7611(3.4%)  57905(26.1%)  118009(53.2%)  36574(16.5%)  774(0.3%)  270(0.1%) | 261 (0.2%)  3423(2.9%)  30372(25.5%)  65161(54.6%)  19652(16.5%)  352(0.3%)  111(0.1%) | 395 (0.4%)  3573 (3.8%)  24841(26.7%)  48405(51.9%)  15459(16.6%)  373(0.4%)  134(0.1%) | 97(1.0%)  615(6.6%)  2692(28.7%)  4443(47.3%)  1463(15.6%)  49(0.5%)  25(0.3%) | *<0.001* |
| Processed meat intake  *No answer*  *Never*  *Less that once a week*  *Once a week*  *2-4 times a week*  *5-6 times a week*  *Once or more daily* | 283 (0.1%)  19180 (9.4%)  70828 (30.8%)  65496 (29.0%)  58393 (26.9%)  6175 (3.0%)  1541 (0.8%) | 73 (0.1%)  9890 (8.3%)  39205 (32.9%)  35608 (29.8%)  30823 (25.8%)  3062 (2.6%)  671 (0.6%) | 161 (0.2%)  8347 (9.0%)  29136 (31.3%)  27245 (29.2%)  24815 (26.6%)  2737 (2.9%)  739 (0.8%) | 49 (0.5%)  943 (10.0%)  2487 (26.5%)  2643 (28.2%)  2755 (29.4%)  376 (4.0%)  131 (1.4%) | *<0.001* |
| Poultry intake  *Never*  *Less than once a week*  *Once a week*  *2-4 times a week*  *5-6 times a week*  *Once or more daily* | 9191(4.1%)  26883(12.1%)  86496 (39%)  95449(43.1%)  3157(1.4%)  401(0.2%) | 4664 (3.9%)  14083(11.8%)  47639 (39.9%)  51254(43.0%)  1475(1.2%)  139(0.1%) | 4026(4.3%)  11433(12.3%)  35434(38.1%)  40425(43.5%)  1472(1.6%)  210(0.2%) | 501(5.4%)  1367(14.7%)  3423(36.7%)  3770(40.4%)  210(2.3%)  52(0.6%) | *<0.001* |
| Beef intake  *Never*  *Less than once a week*  *Once a week*  *2-4 times a week*  *5-6 times a week*  *Once or more daily* | 21046(9.5%)  102586(46.4%)  71629(32.4%)  25508(11.5%)  346(0.2%%)  131(0.1%) | 10568(8.9%)  55931(47.0%)  38932(32.7%)  13496(11.3%)  147(0.1%)  44(0%) | 9304(10.0%)  42755(46.0%)  29766(32.1%)  10796(11.6%)  167(0.2%)  60(0%) | 1174(12.7%)  3900(42.0%)  2931(31.6%)  1216(13.1%)  32(0.3%)  27(0.3%) | *<0.001* |
| Lamb intake  *Never*  *Less than once a week*  *Once a week*  *2-4 times a week*  *5-6 times a week*  *Once or more daily* | 34952(15.8%)  124580(56.4%)  54664(24.8%)  6400(2.9%)  77(0%)  72(0%) | 17797(15.0%)  68565(57.6%)  29417(24.7%)  3111(2.6%)  25(0%)  19(0%) | 15328(16.6%)  51533(55.7%)  22757(24.6%)  2882(3.1%)  41(0%)  36(0%) | 1827(19.8%)  4482(48.5%)  2490(27%)  407(4.4%)  11(0.1%)  17(0.2%) | *<0.001* |
| Pork intake  *Never*  *Less than once a week*  *Once a week*  *2-4 times a week*  *5-6 times a week*  *Once or more daily* | 33858(15.3%)  126548(57.3%)  53138(24.1%)  7091(3.2%)  112(0.1%)  67(0%) | 16927(14.2%)  70229(59.1%)  28324(24.2%)  3368(2.8%)  36(0%)  22(0%) | 15026(16.2%)  51851(56%)  22400(24.2%)  3261(3.5%)  64(0%)  40(0%) | 1905(20.6%)  4468(48.2%)  2414(26.1%)  462(5.0%)  12(0.1%) | *<0.001* |
| Cheese intake  *Never*  *Less than once a week*  *Once a week*  *2-4 times a week*  *5-6 times a week*  *Once or more daily* | 6150(2.9%)  36524(16.9%)  46342(21.5%)  98393(45.6%)  20339(9.4%)  7831(3.6%) | 2689(2.3%)  17817(15.0%)  24409(20.9%)  55415(47.4%)  11839(10.2%)  4381(3.8%) | 2990(3.3%)  16758(18.6%)  19831(22.0%)  39432(43.8%)  7935(8.8%)  3166(3.5%) | 471(5.3%)  1949(21.9%)  2102(23.6%)  3546(39.8%)  565(6.3%)  284(3.2%) | *<0.001* |
| Milk intake  *Never/rarely have milk*  *Yes* | 18183(8.2%)  203623(91.8%) | 9284(7.8%)  110023(92.2%) | 8025(8.6%)  85102(91.4%) | 874(9.3%)  8498(90.7%) | *<0.001* |
| Bread intake (slices/week) | 12.17±8.45 | 12.3±8.3 | 11.96±8.6 | 12.34±9.3 | *<0.001* |
| Salt added to food  *Never/rarely*  *Sometimes*  *Usually*  *Always* | 124102(55.9%)  61096(27.5%)  27010(12.2%)  9671(4.4%) | 68519(57.4%)  32593(27.3%)  13919(11.7%)  4297(3.6%) | 51047(54.8%)  25899(27.8%)  11710(12.6%)  4514(4.8%) | 4536(48.3%)  2604(27.7%)  1381(14.7%)  860(9.2%) | *<0.001* |
| Cereal intake (bowls/week) | 4.76±2.8 | 4.86±2.74 | 4.68±2.84 | 4.21±2.98 | *<0.001* |
| Major dietary changes in the last 5 years  *No*  *Yes because of illness*  *Yes because of other reasons* | 136931(61.7%)  26694(12.0%)  58271(26.3%) | 82028(68.7%)  9603(8.0%)  27701(23.2%) | 51039(54.8%)  13919(14.9%)  28222(30.3%) | 3864(41.2%)  3172(33.8%)  2348(25.0%) | *<0.001* |
| Vitamin supplements  *No*  *Yes* | 146918(66.2%)  74976(33.8%) | 79780(66.9%)  39550(33.1%) | 60999(65.5%)  32181(34.5%) | 6139(65.4%)  3245(34.6%) | *<0.001* |
| Mineral supplements  *No*  *Yes* | 110835(49.9%)  111059(50.1%) | 58420(49.0%)  60910(51.0%) | 47083(50.5%)  46097(49.5%) | 5332(56.8%)  4052(43.2%) | *<0.001* |
| Tea intake (cups/day) | 3.5±2.8 | 3.4±2.7 | 3.5±2.9 | 3.7±3.5 | *<0.001* |
| Coffee intake  *No (decaf)*  *Yes* | 37544(20.8%)  142589(79.2%) | 20087(20.2%)  79213(79.8%) | 15971(21.5%)  58212(78.5%) | 1486(22.3%)  5164(77.7%) | *<0.001* |
| Water intake (glasses/day) | 1.87±0.45 | 2.3±1.95 | 2.45±2.12 | 2.6±2.32 | *<0.001* |
| Breastfed as a baby  *No*  *Yes* | 97254(43.8%)  124629(56.2%) | 50678(42.5%)  68649(57.5%) | 41958(45.0%)  51216(55.0%) | 4618(49.2%)  4764(50.8%) | *<0.001* |
| Maternal smoking around birth  *Do not know*  *No*  *Yes* | 29934(13.7%)  136587(62.5%)  52085(23.8%) | 15770(13.4%)  74656(63.4%)  27341(23.2%) | 12759(13.9%)  56643(61.8%)  22260(24.3%) | 1405(15.3%)  5288(57.6%)  2484(27.1%) | *<0.001* |
| Workplace very noisy  *Rarely/never*  *Sometimes/often* | 20839(37.4%)  34932(62.6%) | 2519(38.3%)  20169(61.7%) | 7953(36.3%)  13940(63.7%) | 367(30.8%)  823(69.2%) | *<0.001* |
| Workplace very cold  *Rarely/never*  *Sometimes/often* | 29335(52.7%)  26371(47.3%) | 17693(54.2%)  14966(45.8%) | 11138(50.9%)  10727(49.1%) | 504(42.6%)  678(57.4%) | *<0.001* |
| Workplace very hot  *Rarey/never*  *Sometimes/often* | 23752(42.6%)  31960(57.4%) | 14383(44.0%)  18280(56.0%) | 8950(40.9%)  12913(59.1%) | 419(35.3%)  767(64.7%) | *<0.001* |
| Workplace very dusty  *Rarely/never*  *Sometimes/often* | 33782(60.7%)  21881(39.3%) | 20203(61.9%)  12421(38.1%) | 12960(59.3%)  8893(40.7%) | 619(52.2%)  567(47.8%) | *<0.001* |
| Workplace with exposure to chemical or other fumes  *Rarely/never*  *Sometimes/often* | 40684(73.1%)  14988(26.9%) | 23961(73.4%)  8679(26.6%) | 15898(72.8%)  5949(27.2%) | 825(69.6%)  360(30.4%) | *0.006* |
| Workplace with asbestos  *Rarely/never*  *sometimes/often* | 49108(88.3%)  6529(11.7%) | 28838(88.4%)  3778(11.6%) | 19242(88.1%)  2595(11.9%) | 1028(86.8%)  156(13.2%) | *0.168* |
| Worked with paints, thinners or glues  *Rarely/never*  *Sometimes/often* | 45787(82.3%)  9876(17.7%) | 26918(82.5%)  5720(17.5%) | 17921(82%)  3922(18%) | 948(80.2%)  234(19.8%) | *0.076* |
| Worked with pesticides  *Rarely/never*  *Sometimes/often* | 53288(95.8%)  2338(4.2%) | 31281(95.9%)  1339(4.1%) | 20884(95.7%)  939(4.3%) | 1123(94.9%)  60(5.1%) | *0.170* |
| Workplace with exposure to diesel exhaust  *Rarely/never*  *Sometimes/often* | 46418(83.5%)  9203(16.5%) | 27362(83.9%)  5244(16.1%) | 18122(83.0%)  3708(17.0%) | 934(78.8%)  251(21.2%) | *<0.001* |

CRP = C-reactive protein, IGF-1 = insulin-like growth factor, SHBG = sex hormone binding globulin

**Supplementary Table 4** Percentage of data variance represented by each of the first 10 Principal Components (PC). After the seventh component, the magnitude of the eigenvalues decreases by approximately 0.1 for each subsequent PC

| PC | Percentage value of data variance represented by each PC |
| --- | --- |
| 1 | 4.8778 |
| 2 | 4.5824 |
| 3 | 3.7059 |
| 4 | 3.5117 |
| 5 | 3.3350 |
| 6 | 2.9426 |
| 7 | 2.6956 |
| 8 | 2.5297 |
| 9 | 2.5074 |
| 10 | 2.4333 |

**Supplementary Table 5** Variables directly and indirectly related to frailty status in terms of angular range across PC 3 and 4, and PC 4 and 5. The first column contains the correlation coefficient between variables and frailty status (FRAILTY_3cat_def), followed by columns containing the number and name of variables directly and indirectly correlated along those PC. See Supplementary Table 1 for notation and acronym explanations

| **Correlation Coefficient** | **N° of directly correlated variables** | **Variables** | | **N° of indirectly correlated variables** | **Variables** |  |
| --- | --- | --- | --- | --- | --- | --- |
| ***Principal Components 3 and 4*** | | | | | |  |
| 0.98 | 8 | Natural env %, CRP, Alcohol previous, Smoking Status, Poultry, Diet_changes5y, Maternal Smoking, Polypharmacy, | | 3 | NO2, NOX, PM10 |  |
| 0.94 | 12 | Age_def, Tea, Natural env %, CRP, Alcohol intake frequency, Alcohol previous, Smoking Status, Smoke exposure_total, Poultry, Diet_changes5y, Maternal Smoking, Polypharmacy | | 3 | NO2, NOX, PM10 |  |
| 0.86 | 14 | Age_def, Pack_years, Tea, Natural env %, CRP, Alcohol intake frequency, Alcohol previous, Smoking Status, Smoke_exposure_total, Poultry, Diet_changes5y, Vitamins Cat, Maternal Smoking, Polypharmacy | | 3 | NO2, NOX, PM10 |  |
| ***Principal Components 4 and 5*** | | | | | | |
| 0.98 | 4 | Natural env %, Alcohol previous, Smoke_exposure_total, Maternal Smoking | 3 | | PM10, PM2, Breastfed | |
| 0.94 | 6 | Pack_years, Natural env %, Alcohol previous, Smoke_exposure_total, Diet_changes5y, Maternal Smoking | 3 | | PM10, PM2, Breastfed | |
| 0.86 | 8 | Pack_years, Natural env %, CRP, Alcol_intake_frequency, Alcohol previous, Smoke_exposure_total, Diet_changes5y, Maternal Smoking | 3 | | PM10, PM2, Breastfed | |

**Supplementary Table 6** Loading values of variables from the first 10 principal components (PC) generated by the principal component analysis (PCA) performed excluding the variable 'FRAILTY_3Cat_def'. This exclusion was made to use the index as the independent variable in principal component regression (PCR) analysis. Each loading represents the contribution of a variable to a specific component. For notation and acronym explanations refer to Supplementary Table 1

| **ACRONYM** | **PC 1** | **PC 2** | **PC 3** | **PC 4** | **PC 5** | **PC 6** | **PC 7** | **PC 8** | **PC 9** | **PC 10** |
| --- | --- | --- | --- | --- | --- | --- | --- | --- | --- | --- |
| **A_d** | 0.022149602 | 0.003226965 | 0.043435513 | 0.09723303 | -0.011631984 | -0.019867507 | 0.22313958 | 0.094720532 | 0.626472866 | -0.045837989 |
| **PY** | 0.096528497 | 0.03656523 | -0.020553253 | 0.036981645 | 0.088979494 | -0.008844052 | 0.010891101 | 0.026360229 | -0.024369836 | 0.218487746 |
| **CV** | -0.03159539 | 0.001035702 | 0.099998941 | 0.103324389 | 0.0331129 | 0.282892162 | -0.023670634 | -0.172634889 | 0.019757101 | 0.160695635 |
| **RV** | -0.099664376 | 0.010485731 | 0.072096953 | 0.063228726 | 0.000706269 | 0.303065037 | -0.100410933 | -0.039797913 | 0.039488302 | 0.218826378 |
| **FF** | -0.200804789 | 0.013409074 | 0.071963081 | 0.075132503 | 0.009983304 | 0.248880179 | 0.119332926 | -0.075223887 | 0.001449348 | 0.094253163 |
| **B** | 0.085815713 | -0.011123183 | -0.02190026 | 0.028051226 | -0.006582113 | -0.00962404 | 0.183337118 | 0.31826434 | -0.033842239 | 0.12209495 |
| **C** | -0.069177762 | -0.027119701 | -0.056055168 | 0.012427558 | -0.145186028 | -0.025587822 | 0.48778124 | -0.104568195 | 0.053219407 | -0.118943738 |
| **T** | -0.00732751 | -0.005910968 | 0.017408307 | -0.017828588 | 0.113987288 | -0.007797797 | 0.187238734 | 0.327433659 | -0.155492727 | -0.03734622 |
| **W** | -0.142696752 | 0.03003912 | 0.104282658 | 0.09214241 | 0.056640058 | 0.186407852 | -0.119031764 | -0.148323915 | 0.04695952 | 0.142312558 |
| **NE** | -0.025513773 | -0.369785725 | 0.00076952 | -0.001423937 | 0.051786238 | -0.010322623 | 0.005518944 | 0.018897992 | -0.003278514 | -0.025997916 |
| **NO2** | 0.039816172 | 0.524897454 | -0.000935363 | 0.001966058 | -0.070150483 | 0.012961575 | 0.025895091 | -0.001537801 | -0.009249222 | -0.032019634 |
| **NOX** | 0.04054934 | 0.519158368 | -0.000247834 | -0.000231885 | -0.059058127 | 0.01143203 | 0.032903682 | 0.014315977 | -0.013685766 | -0.044369054 |
| **PM10** | 0.018036097 | 0.265025346 | 0.001061245 | -0.003875659 | -0.027654872 | -0.01315315 | -0.017500866 | -0.024489183 | 0.005952152 | 0.069268756 |
| **PM2** | 0.038048336 | 0.471220052 | 0.006823059 | -8.38863E-05 | -0.051421306 | 0.004867601 | 0.026275472 | 0.012856931 | -0.007512401 | -0.0324754 |
| **CRP** | 0.011093512 | 0.018324759 | 0.034502454 | -0.005777874 | 0.18377483 | -0.080032225 | -0.081844391 | 0.037325224 | 0.152618468 | 0.196688824 |
| **IGF1** | 0.025035871 | -0.013956636 | -0.037641265 | 0.049155679 | -0.06629185 | 0.070822463 | 0.167802013 | -0.050753088 | -0.326060011 | -0.042149757 |
| **Oe** | -0.213134599 | 0.029227977 | 0.041948924 | -0.181111266 | 0.011922935 | -0.072765801 | -0.168418809 | 0.066192103 | 0.028586205 | -0.103604368 |
| **RhF** | 0.001706008 | 0.014738979 | 0.006843164 | 0.005962105 | 0.001334359 | -0.002279472 | -0.009722016 | 0.043540545 | 0.123181503 | -0.075796681 |
| **SHBG** | -0.154579183 | 0.012359466 | 0.021525662 | -0.147005021 | -0.049843022 | -0.071424344 | -0.096592257 | 0.225880561 | 0.200648595 | -0.195157704 |
| **Ts** | 0.383221707 | -0.04324596 | -0.069099578 | 0.306501499 | -0.048408021 | 0.076274031 | 0.146309866 | -0.101987883 | -0.062071878 | 0.02106558 |
| **VitD** | -0.042566654 | -0.034806589 | 0.042376248 | 0.108614444 | -0.158244078 | -0.068654012 | 0.026033403 | 0.008558813 | -0.099795286 | -0.237132755 |
| **S** | 0.493660163 | -0.050629541 | -0.088285958 | 0.251009726 | -0.065046409 | 0.083230063 | 0.081738053 | -0.041324674 | -0.019901215 | -0.021553754 |
| **Al_s** | 7.88861E-31 | -1.00974E-28 | 0 | 0 | 0 | -6.77626E-21 | 0 | 0 | 0 | 1.11022E-16 |
| **Al_in** | -0.200665513 | 0.056529744 | -0.029712529 | -0.073536728 | 0.403692905 | -0.099361238 | 0.364152954 | 0.134998255 | -0.06215378 | 0.183311609 |
| **Al_6U** | 0 | 0 | 0 | 0 | 0 | 0 | 3.38813E-21 | 0 | 1.73472E-18 | 5.55112E-17 |
| **Al_pr** | 0.000677365 | 0.053963483 | 0.042038547 | 0.141427905 | 0.491204621 | 0.019643711 | 0.118514032 | -0.022856836 | -0.01995812 | -0.061490404 |
| **Sm_S** | 0.158317014 | 0.006850731 | 0.034817394 | 0.218893961 | 0.077343012 | 0.045117056 | -0.298694112 | -0.219924528 | 0.242136249 | -0.211599146 |
| **Sm_e** | 0.121996455 | 0.0632695 | 0.079358748 | 0.047403797 | 0.256853181 | -0.097139551 | -0.210350293 | 0.183127819 | -0.093493786 | 0.067001724 |
| **OiF** | -0.129619853 | -0.005609009 | 0.155113924 | 0.170859083 | -0.107857652 | 0.456811456 | 0.030317537 | 0.187501631 | 0.076333912 | -0.131974328 |
| **NOif** | -0.026008206 | -0.021444363 | 0.191831738 | 0.053827202 | -0.088212871 | 0.337035973 | 0.014938029 | 0.416633339 | 0.087593878 | -0.209246221 |
| **PrM** | 0.282434089 | -0.02027618 | 0.121324252 | 0.053670281 | 0.029127502 | -0.010176236 | -0.074131638 | 0.385232886 | -0.040356796 | 0.055644733 |
| **Plt** | -0.055199405 | 0.008449395 | 0.01507064 | 0.117218081 | 0.069220051 | 0.228765592 | -0.087855071 | 0.094167252 | -0.21989383 | 0.04674277 |
| **Be** | 0.15625558 | -0.022903719 | 0.452723865 | -0.186504921 | -0.023731544 | -0.063616704 | 0.080165946 | -0.082512658 | -0.04661744 | -0.017897498 |
| **La** | 0.149102127 | -0.011909536 | 0.53875774 | -0.191772979 | -0.032880409 | -0.034975038 | 0.077133629 | -0.13500843 | -0.0319917 | 0.01174058 |
| **Pork** | 0.141625564 | -0.017522127 | 0.489003498 | -0.180337733 | 0.012329416 | -0.028874979 | 0.095033357 | -0.080786119 | -0.048485255 | 0.03760519 |
| **Ch** | 0.04102085 | -0.017552173 | -0.016627292 | 0.018510382 | -0.141682773 | 0.060691641 | -0.096832615 | 0.290989932 | -0.101154066 | 0.291398641 |
| **Mi** | 3.28429E-47 | -2.8026E-45 | 0 | 5.87747E-39 | -1.92593E-34 | 2.46519E-32 | -2.52435E-29 | -1.65436E-24 | 0 | 0 |
| **Sa** | 0.069722781 | 0.009753447 | 0.052532062 | -0.010864431 | 0.065595937 | -0.127199531 | -0.345412241 | 0.123129659 | 0.164321063 | 0.093967639 |
| **Co** | -2.18953E-47 | 2.8026E-45 | -5.73972E-42 | 9.18355E-41 | -7.52316E-37 | -4.81482E-35 | 0 | 5.16988E-26 | 0 | -2.11758E-22 |
| **Dc5** | -0.111188452 | 0.034324056 | 0.058655898 | 0.155606947 | 0.359296361 | 0.119697998 | -0.025785813 | -0.135684611 | -0.096342047 | -0.115076755 |
| **ViC** | -0.234874437 | 0.011074148 | 0.200088787 | 0.439566637 | -0.171370007 | -0.335292886 | -0.038468829 | 0.042772519 | -0.114826991 | 0.07863842 |
| **MiC** | -0.243036207 | -0.007862804 | 0.202553943 | 0.436855005 | -0.22922545 | -0.31788282 | 0.004172434 | 0.046194638 | -0.071303178 | 0.042792408 |
| **Bre** | -0.103425613 | 0.008279743 | 0.042078222 | -0.015988176 | -0.126112583 | 0.093931321 | -0.094810239 | -0.0865831 | -0.064950401 | 0.419274753 |
| **Ms** | 0.037433829 | 0.010744447 | 0.010270239 | 0.020031544 | 0.135729891 | -0.021343106 | -0.201393256 | 0.047899603 | -0.354238642 | -0.401762506 |
| **WN** | 0 | 0 | 0 | 0 | 0 | 0 | 0 | 0 | 0 | 0 |
| **WC** | 0 | 0 | 0 | 0 | 0 | 0 | 0 | 0 | 0 | 0 |
| **WH** | 0 | 0 | 0 | 0 | 0 | 0 | 0 | 0 | 0 | 0 |
| **WD** | 0 | 0 | 0 | 0 | 0 | 0 | 0 | 0 | 0 | 0 |
| **WC** | 0 | 0 | 0 | 0 | 0 | 0 | 0 | 0 | 0 | 0 |
| **WAS** | 0 | 0 | 0 | 0 | 0 | 0 | 0 | 0 | 0 | 0 |
| **WPa** | 0 | 0 | 0 | 0 | 0 | 0 | 0 | 0 | 0 | 0 |
| **WPe** | 0 | 0 | 0 | 0 | 0 | 0 | 0 | 0 | 0 | 0 |
| **Wdi** | 0 | 0 | 0 | 0 | 0 | 0 | 0 | 0 | 0 | 0 |
| **Pol** | -0.00821106 | 0.031184356 | 0.152757487 | 0.309313094 | 0.305089144 | -0.129855295 | 0.062559947 | 0.006476794 | 0.183017551 | -0.016549644 |
| **UV** | -0.213904299 | 0.008194646 | 0.100809366 | -0.003806829 | 0.025892854 | 0.118010163 | -0.00730721 | -0.082472805 | -0.074420018 | -0.218391282 |
| **FreS** | 0 | 0 | 0 | 0 | 0 | 0 | 0 | 0 | 0 | 0 |

**Supplementary Table 7** Ranking of variables included in the PC5 by factor loading. For more details on the loading values of each variable across the first 10 principal components (derived from a PCA performed excluding the variable 'FRAILTY_3Cat_def'), see Supplementary Table 6. For notation and acronym explanations refer to Supplementary Table 1

| Alcohol_previous | 0.491204621 |
| --- | --- |
| Alcohol_intake_freq | 0.403692905 |
| Diet_changes_5y | 0.359296361 |
| Polypharmacy | 0.305089144 |
| Smoke_exposure_total | 0.256853181 |
| Minerals_cat | 0.22922545 |
| CRP | 0.18377483 |
| Vitamins_cat | 0.171370007 |
| VitD | 0.158244078 |
| Cereal | 0.145186028 |
| Cheese | 0.141682773 |
| Maternal_smoking | 0.135729891 |
| Breastfed | 0.126112583 |
| Tea | 0.113987288 |
| Oily_fish | 0.107857652 |
| Pack_years | 0.088979494 |
| Non_Oily_fish | 0.088212871 |
| Smoking_status | 0.077343012 |
| NO2 | 0.070150483 |
| Poultry | 0.069220051 |
| IGF1 | 0.06629185 |
| Salt | 0.065595937 |
| Sex | 0.065046409 |
| NOX | 0.059058127 |
| Water | 0.056640058 |
| Natural_env_percent | 0.051786238 |
| PM2 | 0.051421306 |
| SHBG | 0.049843022 |
| Testosterone | 0.048408021 |
| Cooked_vegetables | 0.0331129 |
| Lamb | 0.032880409 |
| Processed_meat | 0.029127502 |
| PM10 | 0.027654872 |
| UV_protection | 0.025892854 |
| Beef | 0.023731544 |
| Pork | 0.012329416 |
| Oestradiol | 0.011922935 |
| Age_def | 0.011631984 |
| Fresh_fruit | 0.009983304 |
| Bread | 0.006582113 |
| Rheumatoid_factor | 0.001334359 |
| Raw_vegetables | 0.000706269 |

**Supplementary Table 8** Accuracy for different combinations of balancing techniques and classifier predictions on training and test sets

| **Training set** | | | | | | |
| --- | --- | --- | --- | --- | --- | --- |
|  | **ROS** | **RUS** | **SMOTE** | **SMOTE Tomek** | **ADASYN** | **CC** |
| **GB** | 0.5328 | 0.5540 | 0.6475 | 0.6506 | 0.6439 | 0.6861 |
| **RF** | 1.0000 | 1.0000 | 1.0000 | 1.0000 | 1.0000 | 1.0000 |
| **XGBoost** | 0.3837 | 0.4614 | 0.4243 | 0.4260 | 0.4188 | 0.6302 |
| **LightGBM** | 0.5627 | 0.6517 | 0.6895 | 0.6898 | 0.6949 | 0.9826 |
| **Testing set** | | | | | | |
|  | **ROS** | **RUS** | **SMOTE** | **SMOTE Tomek** | **ADASYN** | **CC** |
| **GB** | 0.5297 | 0.5143 | 0.6437 | 0.6473 | 0.6420 | 0.6560 |
| **RF** | 0.8003 | 0.5031 | 0.7169 | 0.7146 | 0.7414 | 0.6191 |
| **XGBoost** | 0.3734 | 0.3740 | 0.4214 | 0.4245 | 0.4161 | 0.5882 |
| **LightGBM** | 0.5496 | 0.5148 | 0.6808 | 0.6801 | 0.6901 | 0.9357 |

ROS = Random Over Sampling, RUS = Random Under Sampling, CC = Cluster Centroids, GB = Gradient Boosting, RF = Random Forest.

**Supplementary Fig. 1** Estimates of the principal component (PC) regression coefficients. Each bar represents a PC coefficient in the regression model. For more details on the loading values of each variable across the first 10 PC see Supplementary Table 6. For notation and acronym explanations refer to Supplementary Table 1


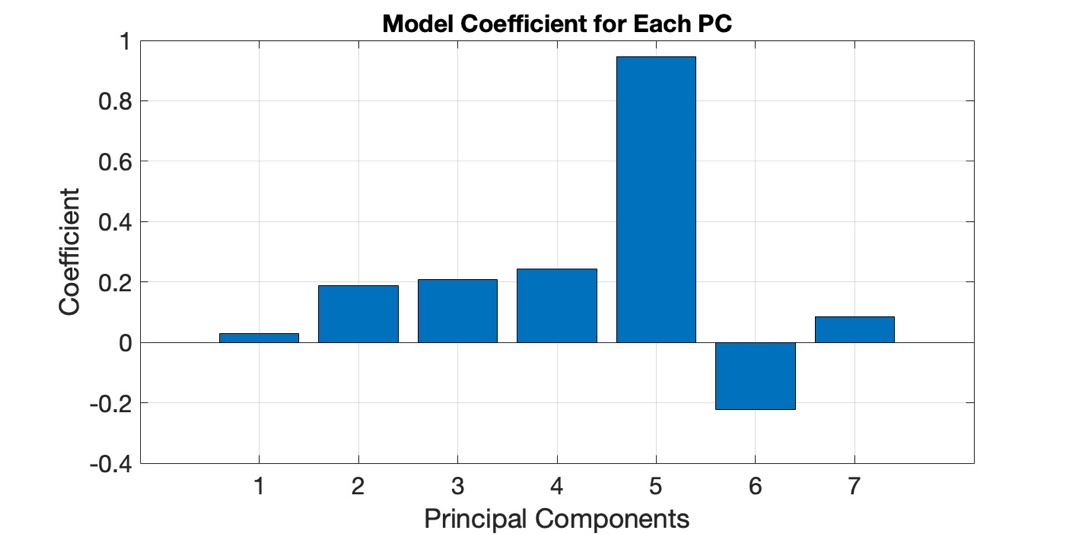

Supplement: Supplementary file 1 — Supplementary file1 (DOCX 398 KB) [file 10522_2025_10242_MOESM1_ESM.docx]
